# Exosomes serve as novel modes of tick-borne flavivirus transmission from arthropod to human cells and facilitates dissemination of viral RNA and proteins to the vertebrate neuronal cells

**DOI:** 10.1371/journal.ppat.1006764

**Published:** 2018-01-04

**Authors:** Wenshuo Zhou, Michael Woodson, Biswas Neupane, Fengwei Bai, Michael B. Sherman, Kyung H. Choi, Girish Neelakanta, Hameeda Sultana

**Affiliations:** 1 Department of Biological Sciences, Old Dominion University, Norfolk, VA, United States of America; 2 Department of Biochemistry and Molecular Biology, University of Texas Medical Branch, Galveston, TX, United States of America; 3 Department of Biological Sciences, The University of Southern Mississippi, Hattiesburg, MS, United States of America; 4 Sealy Center for Structural Biology and Molecular Biophysics, University of Texas Medical Branch, Galveston, TX, United States of America; 5 Center for Molecular Medicine, Old Dominion University, Norfolk, VA, United States of America; 6 Department of Medicine, Division of Infectious Diseases and International Health, University of Virginia School of Medicine, Charlottesville, VA, United States of America; University of California, San Diego, UNITED STATES

## Abstract

Molecular determinants and mechanisms of arthropod-borne flavivirus transmission to the vertebrate host are poorly understood. In this study, we show for the first time that a cell line from medically important arthropods, such as ticks, secretes extracellular vesicles (EVs) including exosomes that mediate transmission of flavivirus RNA and proteins to the human cells. Our study shows that tick-borne Langat virus (LGTV), a model pathogen closely related to tick-borne encephalitis virus (TBEV), profusely uses arthropod exosomes for transmission of viral RNA and proteins to the human- skin keratinocytes and blood endothelial cells. Cryo-electron microscopy showed the presence of purified arthropod/neuronal exosomes with the size range of 30 to 200 nm in diameter. Both positive and negative strands of LGTV RNA and viral envelope-protein were detected inside exosomes derived from arthropod, murine and human cells. Detection of Nonstructural 1 (NS1) protein in arthropod and neuronal exosomes further suggested that exosomes contain viral proteins. Viral RNA and proteins in exosomes derived from tick and mammalian cells were secured, highly infectious and replicative in all tested evaluations. Treatment with GW4869, a selective inhibitor that blocks exosome release affected LGTV loads in both arthropod and mammalian cell-derived exosomes. Transwell-migration assays showed that exosomes derived from infected-brain-microvascular endothelial cells (that constitute the blood-brain barrier) facilitated LGTV RNA and protein transmission, crossing of the barriers and infection of neuronal cells. Neuronal infection showed abundant loads of both tick-borne LGTV and mosquito-borne West Nile virus RNA in exosomes. Our data also suggest that exosome-mediated LGTV viral transmission is clathrin-dependent. Collectively, our results suggest that flaviviruses uses arthropod-derived exosomes as a novel means for viral RNA and protein transmission from the vector, and the vertebrate exosomes for dissemination within the host that may subsequently allow neuroinvasion and neuropathogenesis.

## Author summary

In this study we have demonstrated that cells from the medically important vector tick, secretes exosomes that mediate transmission of tick-borne Langat (LGTV) viruses from arthropod to human and other vertebrate host cells. This study not only provides evidence that suggest tick-borne pathogens use arthropod-derived exosomes for transmission from vector to mammalian cells but also use exosomes for dissemination within the vertebrate host.

## Introduction

Exosomes are small membranous extracellular microvesicles (30 to 250 nm in diameter) of endocytic origin formed in late endosomal compartments (as multivesicular bodies; MVBs) of several different cell types [1–5]. Initially, exosomes were considered as garbage bins to discard the unwanted cellular or molecular components or membranous proteins from reticulocytes [6–9]. Other studies have suggested that exosomes are mere cell debris or apoptotic blebs and signs of cell death [10–12]. Recently, the role of exosomes has been highlighted in important medical research on cancer and autoimmune diseases and they are now recognized as novel therapeutic targets for neurological disorders such as Parkinson’s disease [11,13–16]. Over the past 10 years, exosomes have been given potential biological significance by identifying a variety of their specific roles [3,5,11,17–20]. Exosomes derived from several different cells have been shown to function as signaling related vesicles, transporting cell-specific collections of several proteins, lipids and nucleic acids such as DNA, RNA and microRNA [12,20–28]. Exosomes are released into circulation after the fusion with the plasma membrane and these vesicles serve as mediators of molecular transmission [3,10,18,29]. Cell-derived exosomes have been shown to be important modes of intercellular communication and as transmitters of information over longer distances for e.g., between different tissues or multiple organs [2,15,27,30,31].

Studies have also shown that exosomes are vehicles of transmission for a variety of microorganisms and that some pathogens uses exosomes to manipulate their environments [10,15,32–34]. As an example, malaria parasites, *Plasmodium falciparum*, uses exosomes for communication between infected red blood cells [35]. Hepatitis C virus (HCV), an enveloped RNA virus, associates with exosomes isolated from cell culture supernatants and from infected patients [36,37]. Recent findings of HCV transmission through hepatic exosomes establish infection provides new insight into hepatitis drug discovery [38,39]. Exosomes also function in the transfer of immuno-stimulatory viral RNA from HCV-infected cells to co-cultured plasmacytoid dendritic cells [32]. In addition, exosomes facilitate receptor-independent transmission of replication-competent HCV viral RNA that was found to be in complex with Ago2-miR122-HSP90 in HCV-infected individuals or infected hepatocytes [36]. Interestingly, exosomes have been shown to play dual roles in transmitting Hepatitis A virus (HAV) and HCV, thereby evading antibody-mediated immune responses [40]. It has been demonstrated that Toll-like receptor 3 (TLR-3) activated macrophages release exosomes containing anti-HCV micro (miRNA)-29 family members that suggest a novel antiviral mechanism against HCV infections [41]. Herpes Simplex-1 virus and Epstein-Barr virus also use exosomes for transmission [42,43]. Several studies have suggested exosomes as important players in HIV-1 pathogenesis [33,34,44]. HIV Nef protein secreted in exosomes has been shown to trigger apoptosis in CD4+ T cells and the Gag p17 coding RNA is also targeted to the exosomes [45,46]. HIV-infected cell-derived exosomes have been shown to contain the TAR (Trans-Activation Response Element) miRNA that facilitates production of pro-inflammatory cytokines [47,48]. Moreover, a recent but very highlighting study showed that exosomes from uninfected cells activates the transcription of latent HIV-1 [49].

*Ixodes* ticks transmit several viruses belonging to the family *Flaviviridae* such as tick-borne encephalitis virus (TBEV), Powassan virus (POWV) and Langat virus (LGTV) [50–53]. LGTV is considered as a model biosafety level 2 (BSL2) pathogen to study pathogenesis of TBEV, due to its significant genome homology with the later. Transmission modes of these arthropod-borne flaviviruses (with positive sense single-stranded RNA) are poorly understood [37,54]. Our study shows for the first time that exosomes facilitate transmission of flavivirus RNA and proteins from arthropod to human cells. We have demonstrated that cells from the medically important vector tick, *Ixodes scapularis*, secretes exosomes that mediate transmission of tick-borne LGTV RNA and proteins from arthropod to human. Our study shows the presence of abundant amounts of LGTV RNA and proteins in exosomes isolated from arthropod and neuronal cells. We also found that LGTV-infected tick cell-derived exosomes were capable of transmigrating and infecting naïve human skin keratinocytes (the initial barrier lining the human cells that comes in contact during bites from infected ticks) and human vascular endothelial cells (that comes in contact during arthropod blood feeding). Our data show that vertebrate exosomes mediate transmission of tick-borne LGTV RNA and proteins from infected-brain microvascular endothelial cells (a component of the blood-brain barrier; BBB) to neuronal cells. In addition, we have demonstrated that exosomes containing tick-borne LGTV and mosquito-borne West Nile virus (WNV) facilitate transmission of viral RNA and proteins from one neuronal cell to others suggesting their novel role in neuropathogenesis. Dihydrochloride hydrate, GW4869 (a selective inhibitor for neutral sphingomyelinase; N-SMase, an enzyme that regulates production and release of exosomes), reduced LGTV loads in exosomes and inhibited the transmission of LGTV RNA and proteins in both arthropod and vertebrate host cells. Overall, our study suggests that exosomes are not only the mediators for transmission of arthropod-borne flavivirus RNA and proteins from arthropod to the vertebrate host, but also facilitate dissemination of these infectious RNA and proteins within the vertebrate host, including crossing of BBB cells and allowing neuroinvasion and neuropathogenesis in the Central Nervous System (CNS).

## Results

### Tick cell-derived exosomes contain infectious LGTV replicative RNA, E- and NS1 proteins

**Fig 1:**
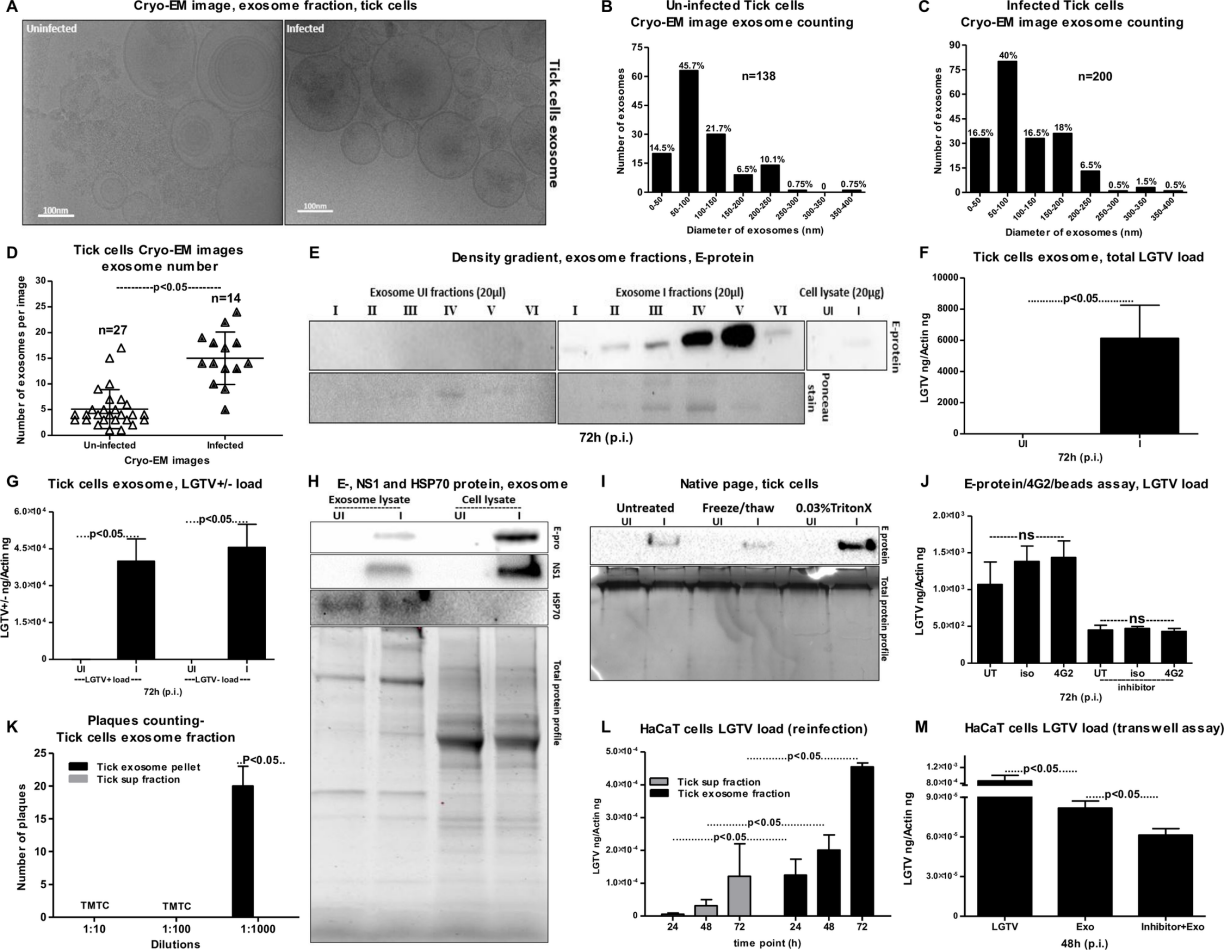
Arthropod exosomes mediate transmission of infectious LGTV RNA and proteins from tick to human cells. (A) Cryo-EM images showing exosomes isolated from uninfected and LGTV-infected (MOI 1; 72 h p.i.), ISE6 tick cells. Scale bar indicates 100nm. Quantification of diameter or sizes for heterogenous population of exosomes from uninfected (B) or LGTV- infected (C) tick cell-derived exosomes. Number of exosomes analyzed were n = 138 (uninfected) and n = 200 (infected) groups. (D) Comparison of exosome numbers per cryo-EM image from uninfected (n = 27) and infected (n = 14) groups is shown. (E) DG-Exos showing presence of LGTV envelope [E]-protein in fractions 1–6. QRT-PCR analysis showing total LGTV loads (F) and levels of LGTV positive-sense strand or negative-sense strand (G) in exosomes isolated from tick cells at 72 h (p.i.), Uninfected cells serve as control. LGTV transcript levels were normalized to tick beta-actin. (H) Immunoblotting analysis showing detection of LGTV E- glycoprotein in exosome fraction and total lysates from whole cells prepared from uninfected (UI) or infected (I) tick cells at 72 h (p.i.). Immunoblot detecting NS1 in both tick-cell derived exosomes and total cell lysates using monoclonal anti-Langat virus NS1 protein from LGTV-infected (MOI 1; 72 h p.i.) is also shown. HSP70 levels indicate enrichment of arthropod exosomal marker in exosome fractions. Uninfected samples and total protein profiles serve as controls. Tick cells (5 x 10^6^) were infected with 1 MOI of LGTV in both QRT-PCR and immunoblotting analysis. I) Native-PAGE followed by immunoblotting analysis showing presence of E-protein from LGTV-infected (MOI 1; 72 h p.i.) or uninfected tick cell-derived exosomes treated with Triton-X-100 (0.03%), or freeze-thaw cycles (3 times freezing at -80°C for 1 h each cycle) or untreated samples held on ice. Coomassie stained gel showing total protein profiles serve as loading control (I). (J) Antibody-beads binding assay performed on LGTV infected (MOI 1) tick cell-derived exosomes (collected from tick cells either untreated or treated with GW4869; 5 μM, exosome inhibitor) showing no differences in Langat-E protein loads between 4G2 or isotype and untreated samples. (K) Quantitative assessment of number of plaques in exosomal and supernatant fractions is shown. TMTC indicates “too many to count”. (L) Viral re-infection kinetics as determined by the presence of total LGTV loads in HaCaT cells (1 x 10^5^ cells) at different time points treated with exosomes (20 μl) or supernatant fractions (400 μl) prepared from 72 h p.i. LGTV-infected tick cells are shown. (M) Viral loads at 48 h p.i. was determined by the presence of LGTV in a transwell assay performed with 1 x 10^5^ tick cells (in upper chamber) or 1 x 10^5^ HaCaT cells (in lower chamber) treated with tick exosome fraction (20 μl, in upper chamber) for 4 h in the presence or absence of 5 μM exosome inhibitor GW4869. Tick cells infected with LGTV laboratory stocks were used as controls. LGTV transcript levels were normalized to human beta-actin in (L) and (M). P value determined by Student’s two-tail *t* test is shown. Representative data is shown from three independent experiments.

Despite the significance of ticks as important medical vectors, we know little about the transmission modes of tick-borne viruses and other tick-borne pathogens to the vertebrate host. We first analyzed whether tick cells secrete extracellular vesicles (EVs) and exosomes and if tick-borne flaviviruses use those exosomes as modes of pathogen transmission. LGTV, a flavivirus closely related to TBEV, readily infected *Ixodes scapularis* ISE6 tick cells, with increased viremia at 72 h post-infection (p.i.) (**S1A Fig**), similar to the viral infection kinetics observed in Vero cells (**S1B Fig**). We selected 72 h p.i. as the time point for the isolation of exosomes from tick cells due to the higher viral loads. First, we isolated exosomes by density gradient centrifugation technique using OptiPrep (DG-Exo-iso) as described in [55]. This isolation method used in our settings with a floor ultracentrifuge unit is shown as a schematic representation in (**S1C Fig**). Exosomes were also independently isolated by differential ultracentrifugation with slight modifications and longer spin times for 155 minutes (**S2 Fig**) [22,29,32,56,57]. We also isolated arthropod-derived exosomes using commercially available exosome isolation reagent following manufacturer's instructions (Invitrogen/ThermoScientific). Notably, all preparations contained 30 to 200 nm vesicles and these techniques have been used extensively in several studies. Cryo- Electron Microscopy (cryo-EM) performed on tick cell-derived exosomal fractions showed the presence of purified arthropod exosomes with the size range of 30 to 200 nm in diameter (**Fig 1A**), similar to exosomes isolated from mammalian cells [1–3]. Exosomes isolated from arthropod cells showed a heterogenous population of vesicles in the cryo-EM analysis. In order to understand such heterogeneity in exosome populations, we did quantitative analysis using images collected from both uninfected and LGTV-infected tick cell-derived exosomes. We noted that majority of the exosomes were of sizes between 50–100 nm in both uninfected and infected groups (**Fig 1B and 1C**). However, exosomes of other sizes 100–150 and 150–200 nm were evenly distributed in infected group when compared to the uninfected group (**Fig 1B and 1C**). Fewer vesicles from sizes of 200–250 nm were slightly more in uninfected (10.1%) in comparison to the infected group (6.5%). The large exosomes were very few and were from 0–1.5% in both the uninfected and infected groups (**Fig 1B and 1C**). Counting of exosomes per image showed higher number of exosomes in LGTV-infected (n = 14) in comparison to the uninfected (n = 27) group (**Fig 1D**). This data suggested that LGTV-infection (72 h p.i.) might enhance the production and/or release of exosomes.

The OptiPrep (DG-Exo-iso) method yielded purified exosomes in six different fractions. Immunoblotting analysis (with highly cross-reactive 4G2 monoclonal antibody that recognizes the viral Envelope (E)- protein) of these fractions (20 μl) showed presence of LGTV E-protein in all six fractions but enriched amounts of E-protein were present in fractions four and five in comparison to the other fractions (**Fig 1E**). These results correlated with the size analysis data (**Fig 1B, 1C and 1D**). Enhanced detection of LGTV-E protein in fractions four may correspond to the 50–100 nm (fraction 4) size exosomes that are highly populated (**Fig 1E**). As expected, we did not detected E-protein in the fractions from uninfected control. Total cell lysates (20 μg) from uninfected and LGTV-infected groups were used as internal controls to compare the amounts of E-protein detected in the 20 μl of different fractions used (**Fig 1E**). The PonceauS images showing the protein profile serve as control (**Fig 1E**). Quantitative Real-Time PCR (QRT-PCR) analysis revealed presence of LGTV total mRNA in exosomes isolated from infected tick cells (**Fig 1F**). The copy numbers of viral RNA in exosomes derived from LGTV-infected (72 h p.i.) tick cells is shown in (**S3A Fig**). In addition, we also determined the presence of both positive and negative sense LGTV RNA strands in tick cell-derived exosomes (**Fig 1G**). LGTV mRNA was also evident in exosomes from tick cells cultured and infected in exosome-free FBS medium (with no cross-contaminating bovine exosomes present in regular commercial FBS), further suggesting the presence of viral RNA in tick cell-derived exosomes (**S3B Fig**). Presence of LGTV E-protein in tick cell-derived exosomes was further recognized by SDS-PAGE followed by immunoblotting with 4G2 antibody (**Fig 1H**). Higher E-protein loads were detected (at ~50kDa) in total cell lysates in comparison to exosomal preparations (**Fig 1H**). Immunoblotting with monoclonal anti-Langat virus NS1 (Clone 6E11) antibody (obtained from BEI resources) also showed the presence of NS1 in both tick cell-derived exosomes and total cell lysates (**Fig 1H**). Although, higher NS1 protein loads were evident in total lysates, but the presence of NS1 in tick-cell derived exosomes (**Fig 1H**) further confirmed that these arthropod exosomes contain LGTV proteins. Remarkably, we also detected the presence of tick HSP70 (heat-shock cognate protein 70, a specific exosomal marker in mammalian cells) in exosomal lysates (**Fig 1H**). No differences were noted in HSP70 loads between uninfected and infected exosomal lysates (**Fig 1H**). Presumably due to low amount in cell lysates, no HSP70 was detected in the tested condition (**Fig 1H**). Total protein lysates prepared from same batch of uninfected or LGTV-infected tick cell-derived exosomes or from whole tick cells served as loading control for all immunoblots (**Fig 1H**). It was noted that some of the bands in the total protein profile gel were enhanced in LGTV-infected tick exosome lysates in comparison to the uninfected controls (**Fig 1H**).

Furthermore, native-PAGE followed by immunoblotting with 4G2 antibody, showed enhanced levels of LGTV E-protein (at <250kDa; in native state) in exosomes treated (30 min, RT) with Triton-X-100 (a detergent that lyses the exosomal lipid bilayer membranes) in comparison to the exosomes treated for three rounds of freeze-thaw cycle (1 h, at -80°C) or the untreated exosomes held at 4°C (**Fig 1I**). Total protein lysates prepared from uninfected or LGTV-infected tick cell-derived exosomes with similar treatments served as controls in this immunoblotting analysis (**Fig 1I**). Detection of LGTV E-protein inside exosomes (but not outside in the PBS suspensions) was further analyzed by E-protein-4G2-antibody-beads binding assay as described in methods. No significant (P>0.05) differences in viral loads were observed in LGTV-infected (72 h p.i.) exosome samples that were either untreated or treated with 4G2 antibody (that binds to LGTV E-protein) or relevant isotype control antibody (**Fig 1J**). Similar results were obtained with LGTV-infected exosomal preparations derived from GW4869 inhibitor treated tick cells collected at 72 h p.i., (**Fig 1J**). Native-PAGE and the beads assay clearly suggest that exosomes contain viral RNA and proteins inside exosomes. To further evaluate if viral E-protein is indeed (totally) inside the exosomes, we performed the protease-resistance assay with Proteinase K that generally digest proteins in all biological samples. We found that treatment with Proteinase K (0.5 μg/μl or 50 μg/ml, 15 min at 37 ºC) at typical and suggested working concentrations (50–100 μg/ml) digested all proteins (**S3C Fig**). We detected E-protein in untreated infected samples but not in treated infected samples. Uninfected samples either treated or untreated served as internal controls (**S3C Fig**). The Ponceau S stained blot showed no proteins in infected or uninfected proteinase K-treated samples (**S3C Fig**). During isolation of tick exosomes, pellet fraction (containing exosomes) and supernatant fraction (generated after pelleting exosomes and before PBS wash; See **S2 Fig**) was tested in plaque assays to determine infectivity and replication of viral RNA and titers as described in methods. Plaque assays performed with the tick cell-derived exosome pellet fractions yielded plaques at dilutions of 1:10 and 1:100 that were too numerous to count, and around 20–22 plaques at a dilution of 1:1000 (**Fig 1K and S3D and S3E Fig**). No plaques were detected in plates where Vero cells were treated with the supernatant fractions at any tested dilution (**Fig 1K and S3D and S3E Fig**). Plaque assays indicated the presence of infectious viral RNA or proteins in LGTV-infected exosomes that resulted in high loads of LGTV in Vero cells (6.6 x 10^4^ pfu/ml) and increased formation of viral plaques. Plaque assays further confirmed that tick cell-derived exosomes contain LGTV RNA and proteins capable of replication and forming viable plaques that are highly infectious to mammalian cells (**Fig 1K and S3D and S3E Fig**). No detection of viral plaques in the supernatant fractions suggests presence of abundant amounts of LGTV RNA and proteins in exosomes (**Fig 1K and S3D and S3E Fig**). Overall, these results suggest that majority of the LGTV RNA and proteins exit tick cells via exosomes and that exosomes could mediate transmission of these and possibly the other closely related viruses such as TBEV and POWV.

### Infectious LGTV RNA is transmitted to human cells via tick exosomes

As tick-borne viruses (including TBEV, LGTV and Powassan virus) are transmitted by an infected tick bite to the vertebrate hosts, we tested whether exosomes isolated from LGTV-infected tick cells are infectious to human cells. In an infection kinetics assay, LGTV readily infected human keratinocytes (HaCaT cells) at all tested time points (24, 48 and 72 h p.i.) and there were no changes in viral loads at different times p.i. (**S3F Fig**). Infection of HaCaT cells with exosome fraction prepared from LGTV-infected tick cells (72 h p.i.) showed significantly (P<0.05) increased levels of viral loads at 72 h p.i. in comparison to HaCaT cells treated with supernatant fractions prepared from 72 h post-infected-tick cells (**Fig 1L**). Tick cell-derived exosomes containing LGTV grown in the presence of exosome-free FBS medium were also found to be infectious to HaCaT cells (**S3G Fig**). However, LGTV was not detectable in HaCaT cells (grown in exosome-free FBS medium) treated with the supernatant fractions (**S3G Fig**). Our data also showed that LGTV (laboratory viral stocks, prepared from Vero cells) was capable of infecting human vascular endothelial (HUVEC) cells with no differences in viral loads at 24 h p.i. in comparison to later tested time points (48 and 72 h p.i.) (**S3H Fig**). HUVEC cells treated with exosomes-containing-LGTV showed significantly (P<0.05) increased viral loads at 48 h p.i. in comparison to the cells treated with supernatant fractions, suggesting that LGTV RNA is enriched in exosomes (**S3I Fig**). We then performed transwell assays (as described in the methods) to test whether tick exosomes mediate transmission of LGTV from infected tick cells (plated in upper inserts) to uninfected/naïve human keratinocytes (seeded into the lower well). We found that tick cells treated with infected tick-cell-derived exosomes (that were isolated from independent batch of LGTV-infected tick cells) readily transmitted infectious exosomes to uninfected HaCaT cells (**Fig 1M**). However, upon incubations with tick cell-derived exosomes collected from GW4869 (5 μM) treated cells, significantly (P<0.05) reduced transmission of viral RNA to HaCaT cells was noted (**Fig 1M**). Infection of arthropod cells with laboratory virus stocks with known titers (MOI 1) served as control in this assay (**Fig 1M**). Taken together, these results suggest that LGTV infectious RNA and proteins are transmitted to human cells via arthropod exosomes.

### LGTV RNA is transmitted from brain endothelial barrier cells to neuronal cells via exosomes

**Fig 2:**
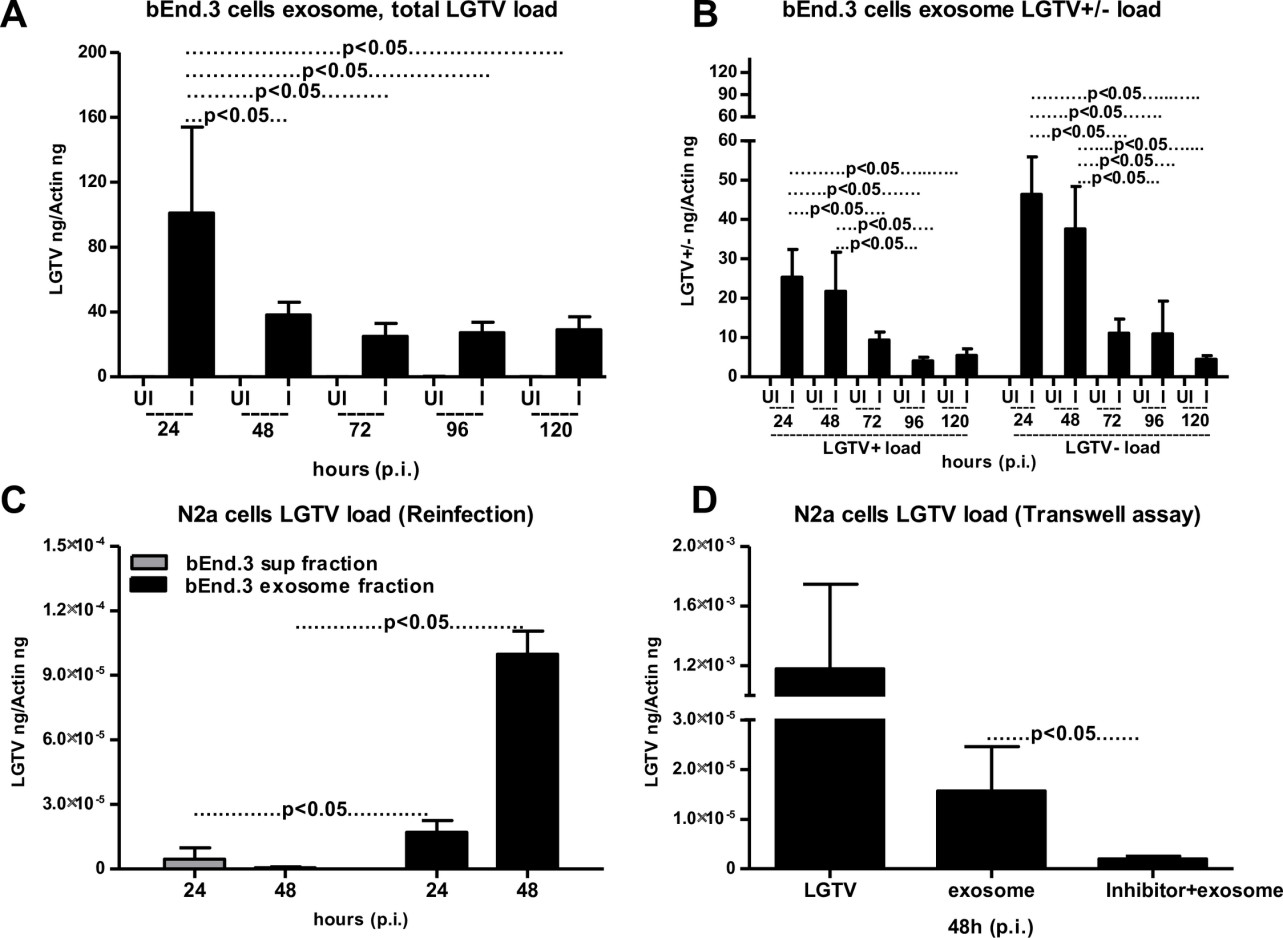
Endothelial cell-derived exosomes mediate transmission of LGTV RNA from bEnd.3 cells to N2a neuronal cells. The bEnd.3 cells (1 x 10^5^) were infected with 6 MOI of LGTV. QRT-PCR analysis showing total LGTV loads (A) or levels of LGTV positive-sense strand or negative-sense strand (B) in exosomes isolated from bEnd.3 cells at different time points (24, 48, 72, 96 and 120 h p.i.). (C) Viral loads were determined after 24 h p.i. by the presence of LGTV in N2a cells (1 x 10 ^5^) treated with LGTV containing exosomes (20 μl) or supernatant (400 μl) fractions prepared from 24 or 48 h p.i. of bEnd.3 cells. (D) Viral loads at 48 h p.i. of N2a cells (plated in lower chamber) as determined by the presence of LGTV in a transwell assay performed with 1x 10^5^ of bEnd.3 cells treated with 20 μl brain endothelial cell-derived exosomes (in upper chamber) for 4 h in the presence or absence of exosome inhibitor (5 μM) (in upper chamber) or infected with LGTV laboratory virus stocks is shown. LGTV transcript levels were normalized to mouse beta-actin. P value determined by Student’s two-tail *t* test is shown. Representative data is shown from three independent experiments.

Upon transmission to the vertebrate host, arthropod-borne neurotropic encephalitis viruses are known to first replicate in the blood and peripheral tissues (spleen and liver), cross the BBB and invade the CNS [58,59]. We used mouse brain-microvascular endothelial cells (bEnd.3 cells; that constitutes the BBB) to test whether LGTV infectious RNA and viral proteins are transmitted to neuronal cells via bEnd.3 cell-derived exosomes. LGTV readily infected and replicated in bEnd.3 cells at all tested time points (48 and 72 h p.i.) (**S4A Fig**). In addition, we found that the viral loads in brain endothelial cells were not significantly different over the infection period as revealed by the viral loads at much later time points (96 and 120 h p.i.) (**S4B Fig**). QRT-PCR analysis revealed significantly (P<0.05) increased viral burden and copy numbers in exosomes isolated from bEnd.3 cells at 24 h p.i. in comparison to the other tested time points (48, 72, 96 and 120 h p.i.) (**Fig 2A and S4C Fig**). We also detected higher loads of LGTV positive and negative sense RNA strands at 24 and 48 h p.i., in comparison to the other tested time points (72, 96 and 120 h p.i.) (**Fig 2B**). LGTV infected and replicated in neuronal cells (mouse N2a cells) in a time-dependent manner with peak level of infection at 72 h p.i. (**S4D Fig**). N2a cells were then infected with bEnd.3 cell-derived exosomes collected at different time points (24 and 48 h p.i.). LGTV RNA and proteins containing exosomes from bEnd.3 cells were found to be infectious to N2a cells with peak level of infection observed with exosomes isolated from endothelial cells at 48 h (p.i.) (**Fig 2C**). N2a cells treated with supernatant fractions (collected at the indicated time points) derived from endothelial cells resulted in significantly (P<0.05) lower viral loads in comparison to the treatments with exosome fractions isolated from the bEnd.3 cells (**Fig 2C**). Transwell assays performed with exosomes isolated from LGTV-infected-brain endothelial cells showed transmission of viral RNA and proteins from bEnd.3 cells (plated in upper inserts) to uninfected/naïve N2a cells seeded in the lower well (**Fig 2D**). Presence of exosome inhibitor significantly reduced transmission of LGTV infectious RNA from bEnd.3 cell-derived exosomes to N2a cells (**Fig 2D**). Infection of bEnd.3 cells with laboratory virus stocks with known titers (6 MOI) showed transmission of LGTV to N2a cells (by crossing the membrane barriers in transwell plates) and served as control in this assay (**Fig 2D**). These results suggest that exosomes derived from brain-endothelial cells are perhaps the mediators for BBB permeability (crossing of infectious exosomes from infected-endothelial cells lining the BBB and transmission to the neuronal cells) that may facilitate neuroinvasion of tick-borne LGTV and possibly TBEV and POWV.

### Neuronal cell-derived exosomes contain LGTV infectious RNA and proteins

**Fig 3:**
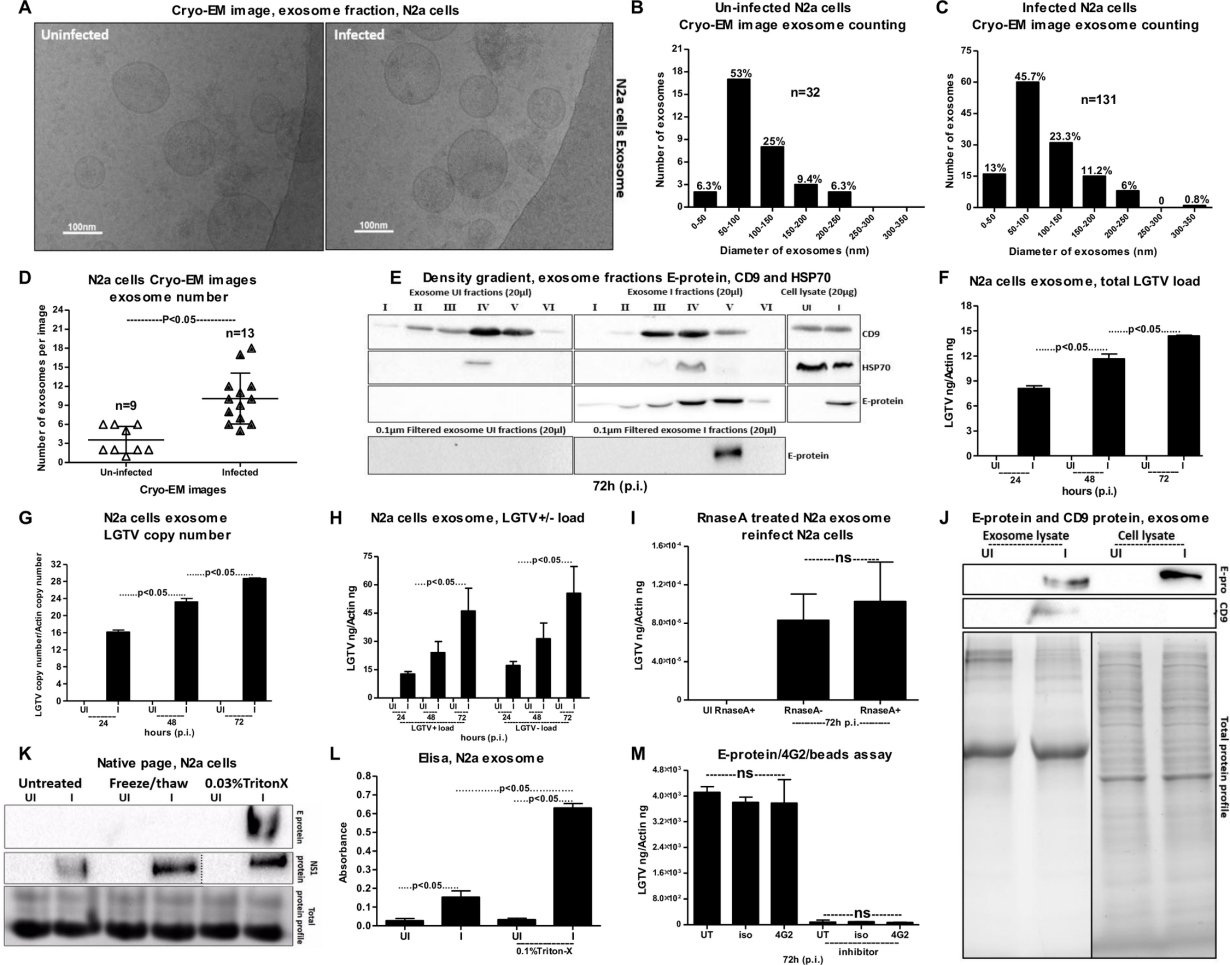
Detection of LGTV RNA and proteins in exosomes isolated from N2a neuronal cell line. (A) Cryo-EM images showing exosomes isolated from uninfected and LGTV-infected (MOI 6; 72 h p.i.), N2a cells (1 x 10^7^). Scale indicates 100nm. Quantification of diameter or sizes for heterogenous population of exosomes from uninfected (B) or LGTV- infected (C) N2a cell-derived exosomes. Number of exosomes analyzed were n = 32 (uninfected) and n = 131 (infected) groups. (D) Comparison of exosome numbers per image from uninfected (n = 9) and infected (n = 13) groups is shown. (E) DG-Exos showing presence of enhanced LGTV envelope [E]-protein loads in fractions 1–6. Fractions 3–5 showed enriched amounts of exosomal markers CD9 and HSP70. E-protein detection in fraction 5 in 0.1 μm filtered samples processed for OptiPrep DG-isolation is shown. QRT-PCR analysis showing levels of total LGTV loads (F), copy numbers (G) and LGTV positive-sense strand or negative-sense strand (H) in exosomes isolated from N2a cells at different time points. N2a (1 x 10^5^) cells were infected with 6 MOI of LGTV, and LGTV loads were analyzed at 72 h p.i. (I) Treatment of LGTV-infected (72 h p.i.) N2a cell-derived exosomes with RNase A is shown. The uninfected samples treated with RNase serve as control. LGTV transcript levels were normalized to mouse beta-actin. (J) Immunoblotting analysis showing detection of LGTV E glycoprotein and mammalian exosomal marker CD9 in exosome fractions and total lysates from whole cells prepared at 48 h p.i. from 2 x 10^6^ uninfected (UI) or infected (I) N2a cells. Stain-free gels showing total protein profiles serve as the loading control. (K) Native-PAGE followed by immunoblotting analysis showing presence of LGTV E- and NS1 proteins from LGTV-infected (MOI 1; 72 h p.i.) or uninfected N2a cell-derived exosomes treated with Triton-X-100 (0.03%), or freeze-thaw cycles (3 times freezing at -80°C for 1 h each cycle) or untreated, held on ice. Coomassie stained gel image showing total protein profiles serve as a loading control. (L) ELISA performed on uninfected or LGTV-infected (MOI 6; 72 h p.i.) N2a cell-derived exosomes either untreated or treated with Triton-X-100 (0.1%). (M) Antibody-beads binding assay performed on LGTV infected (MOI 1, 72 h p.i.) N2a cell-derived exosomes (collected from N2a cells either untreated or treated with GW4869; 5 μM, exosome inhibitor) showing no differences in LGTV-E protein loads between 4G2 or isotype and untreated samples.

Upon entry in to the brain, tick-borne neuroinvasive viruses (such as TBEV) infects neuronal cells [60]. To test whether transmission of these viruses within the brain from one neuronal cell to another is mediated by exosomes; we first infected N2a cells with LGTV (**S4C Fig**). Cryo-EM showed the presence of purified exosome preparations from neuronal cell-derived exosomal fractions with the size range of 30 to 200 nm in diameter (**Fig 3A**), similar to exosomes isolated from tick cells. Also, we isolated exosomes by precipitation using the commercially available kit isolation reagent following the manufacturer’s protocol (**S5A Fig**). Cryo-EM images (generated using this method) showed the presence of purified exosome preparations from neuronal cell-derived exosomal fractions with the similar size range of 30 to 200 nm in diameter (**S5B Fig**). Like arthropod exosomes, neuronal cell-derived exosomes also showed a heterogenous population of vesicles. In a very similar way, we did quantitative analysis using cryo-EM images collected from both uninfected and LGTV-infected N2a cell-derived exosomes. Majority of these exosomes were also of sizes between 50–100 nm in both uninfected and infected groups (**Fig 3B and 3C**). Smaller exosomes of sizes 0–50 nm were of slight higher percentages in infected exosomes when compared to the uninfected group (**Fig 3B and 3C**). Fewer vesicles from sizes of 150–200 (9–11%) or 200–250 (6.3%) were found in both infected and uninfected groups. Less than 1% of larger vesicles (250–350 nm sizes) were found in infected group (**Fig 3B and 3C**). Counting of exosomes per image showed higher number of exosomes in LGTV-infected (n = 13) in comparison to the uninfected (n = 9) group (**Fig 3D**). This data suggested that LGTV-infection (72 h p.i.) might enhance the production of exosomes. The OptiPrep density gradient exosome separation (that separates exosomes from viruses and large microvesicles) yielded purified exosomes at six different fractions. Immunoblotting analysis (using 4G2 antibody) of these fractions (20 μl) showed presence of LGTV E-protein in all fractions but enriched amounts of E-protein were present in fractions four and five in comparison to the other fractions (**Fig 3E).** We did not detect E-protein in the fractions from uninfected control. The cell lysates (20 μg) from uninfected and infected groups were used as controls to compare the amounts of E-protein detected in the different fractions volume (**Fig 3E**). Immunoblotting with anti-HSP70 antibody detected enriched amounts of HSP70 (exosomal marker) in fourth fraction of both uninfected and infected samples (**Fig 3E**). HSP70 levels were also detected in three and five of infected fractions but not in uninfected fractions (**Fig 3E**). In addition to the HSP70, we also analyzed the CD9 (a protein enriched in the mammalian cell-derived exosomes and recognized as exosomal marker) levels in uninfected and infected fractions. CD9 was detected in all six of the uninfected fractions in an increasing manner, with higher levels in fractions four and five (**Fig 3E**). However, CD9 was detected in 2–5 of infected fractions with higher-level detection in fractions three and four (**Fig 3E**). It was interesting to note that LGTV E-protein was enhanced in similar exosomal fractions (fractions 3–5) that had enhanced loads of both HSP70 and CD9, suggesting that infectious exosomes in fraction four have higher levels of exosomal markers. OptiPrep DG-isolation of exosomes using 0.1 μm filter (culture supernatants were filtered before concentration and processing for gradient steps) detected E-protein also in the fraction 4, suggesting that these infectious exosomes have sizes of 50–100 nm (**Fig 3E**). This data also correlated with the quantitative analysis from cryo-EM images. In order to address, where the intact LGTV particles may run on the parallel gradients, we performed OptiPrep DG-isolation on the laboratory stocks of LGTV (prepared in Vero cells, collected at 7–14 days post-infection and stored at -80°C). We noted a differential pattern in E-protein loads when density gradients were performed on LGTV-infected exosomal fractions from N2a cells (**Fig 3E**) or on LGTV laboratory stocks containing viruses (**S5C Fig**). An enhanced E-protein signal was detected in fraction 6 (indicating presence of virions in this fraction) and not in fraction 5. Detection of E-protein in fractions 4, 3 and 2 from the laboratory virus stock suggested the presence of infectious exosomes containing viral E-protein (**S5C Fig**). This data indicated that the viral stocks are not just the virions but are perhaps mixtures of infectious exosomes containing viral E protein.

Upon LGTV infection of N2a cells, exosomes were collected at different time points (24, 48, 72 h p.i.) and analyzed for viral loads. QRT-PCR analysis revealed an increased total viral RNA load and copy numbers at 72 h p.i. in comparison to the other tested time points (24 and 48 h p.i.) (**Fig 3F and 3G**). Both positive- and negative-sense RNA was detected at higher levels in the exosomes isolated from N2a cells at 72 h p.i. in comparison to the other tested time points (**Fig 3H**). Exosomes collected from the kit reagent also yielded similar results with increased LGTV loads in exosomes (**S5D Fig**). Next, we addressed the possibility that viral RNA could be binding to the outside of the exosomes and may be transmitted to the recipient cells. In order to test this possibility, we treated freshly prepared LGTV-infected (72 h p.i.)- N2a cell-derived exosomes with RNase A (5 μg/ml, for 15 min, at 37°C). We did not find any differences in LGTV loads from infected treated or untreated groups (**Fig 3I**). The uninfected group treated with RNase A was kept as internal control (**Fig 3I**). In addition, we treated freshly derived exosomes isolated from LGTV-infected N2a cells, with Triton-X-100 (0.1%; for 45 min, at RT), followed by treatments with RNaseA (5 μg/ml, for 15 min, at 37°C). QRT-PCR analysis showed that exosomes treated with both Triton-X-100 and RNaseA has lower LGTV loads in comparison to exosomes not treated with RNaseA (**S5F Fig**). Immunoblotting analysis further suggested the presence of LGTV E-protein in the exosomes isolated from N2a cells (**Fig 3J**). The E-protein loads were one-or two-fold higher in total lysates in comparison to the exosomal lysates derived from N2a cells (**Fig 3J**). Reduced molecular mass of LGTV E protein was found in exosomes derived from N2a cells in comparison to the total lysates (**Fig 3J**), suggesting a possible de-glycosylation of the viral E protein in neuronal cell-derived exosomes. We found similar de-glycosylation of the viral E protein in immunoblots performed on laboratory virus stocks (**S5E Fig**). A high level of CD9 was detected in the LGTV-infected N2a cell-derived exosomes in comparison to low levels in the uninfected control and the total cell lysates prepared from LGTV-infected or uninfected N2a whole cells (**Fig 3J**). Total protein lysates used in the immunoblot analysis served as loading control (**Fig 3J**). Enhanced levels of LGTV- E protein in neuronal exosomes treated with Triton-X-100 (0.03%; for 30 min, RT) in comparison to the exosomes treated after freeze-thaw cycle (thrice frozen and thawed at -80°C) or untreated exosomes held at 4°C was detected by native-PAGE followed by immunoblotting with 4G2 antibody (**Fig 3K**). We noticed that E-protein was detected at higher molecular mass (<250kDa) in neuronal exosomes when samples were processed for native-PAGE analysis under non-reducing and non-denaturation conditions. Detection of NS1 protein in independent samples at the similar molecular mass suggests presence of other LGTV proteins or polyprotein in exosomes (**Fig 3K**). Exosomes derived from uninfected N2a cells served as control (**Fig 3K**). Total protein lysates prepared from uninfected or LGTV-infected neuronal cell-derived exosomes after freeze-thaw or Triton-X-100 treatments or untreated samples served as loading control (**Fig 3K**). ELISA corroborate results of the native-PAGE, where higher loads of LGTV E-protein were detected when exosomes were treated with 0.1% of Triton-X-100 in comparison to untreated exosomal fractions (**Fig 3L**). Lower level of E-protein in LGTV-infected untreated neuronal exosomes was considered as background signal due to non-specific antibody binding (**Fig 3L**).

**Fig 4:**
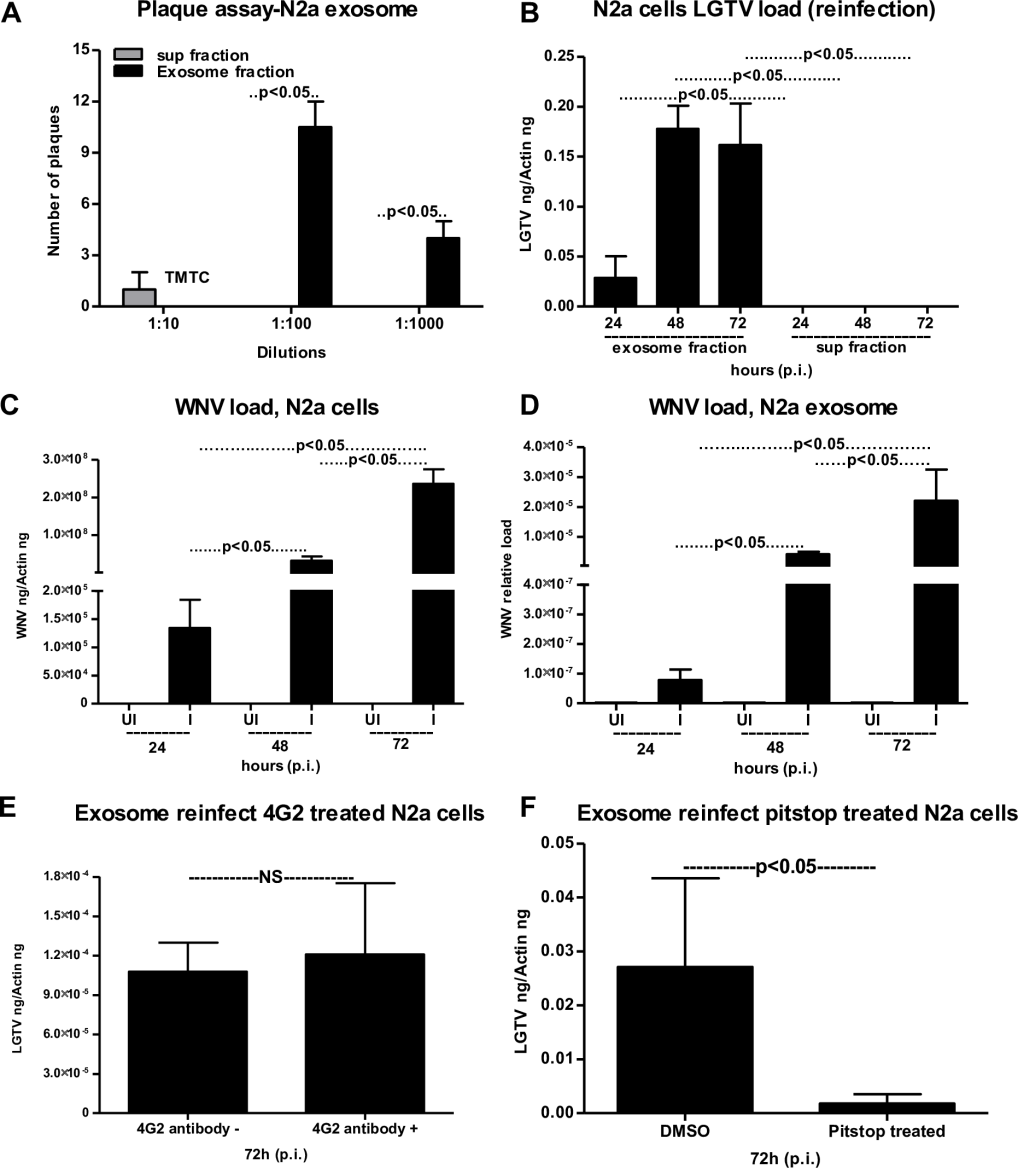
Transmission of LGTV through infectious exosome to naïve cells is clathrin dependent. (A) Quantitative assessment of number of plaques from exosomal and supernatant fractions is shown. TMTC indicates “too many to count”. (B) QRT-PCR analysis for the LGTV loads in fresh N2a (1 x 10 ^5^) cells at 24 h p.i., infected with exosomes (20 μl) or supernatant (400 μl) fractions prepared from 24, 48 and 72 h p.i. (C) Mouse N2a cells showing infection kinetics with WNV (MOI 5) at different times (24, 48, 72 h) p.i., WNV *E* gene transcript levels were normalized to mouse beta-actin. (D) Relative loads of WNV in infected N2a cell-derived exosomes collected at 24, 48, 72 h p.i. LGTV loads (72 h p.i.) in N2a cells treated with either 4G2 antibody (5 μg for 4 h) (E) or Pitstop-2 clathrin inhibitor (F) (treated 30 μM for 15 min at 37°C) and infected with LGTV-containing (72 h p.i.) N2a cell-derived exosomes is shown. Infected but untreated samples serve as control in (E). Infected but DMSO-treated serve as control in (F). LGTV transcript levels were normalized to mouse beta-actin. P value determined by Student’s two-tail *t* test is shown. Representative data is shown from at least three independent experiments.

Furthermore, we analyzed the presence of E-protein inside neuronal exosomes by a 4G2-antibody-coated bead-binding assay as described in methods (**Fig 3M**). No significant (P>0.05) differences in viral loads were observed in LGTV-infected (72 h p.i.) neuronal exosome samples that were untreated/treated with either 4G2 antibody or isotype control antibody (**Fig 3M**). GW4869 inhibitor treated exosomes from LGTV-infected neuronal cells collected at 72 h p.i., followed by treatments with either 4G2 or isotype control also showed no significant (P>0.05) differences in viral load in comparison to untreated samples (**Fig 3M**). However, a significant decrease in LGTV loads were observed in the inhibitor treated group in comparison to no-inhibitor treated group (**Fig 3M**). In addition, we found that exosomes treated with Proteinase K (100 μg/μl, 15 min at 37°C) may be digested all proteins on the surface, thereby, lysing the vesicles and allowing degradation of the exosomal luminal proteins (**S5G Fig**). We detected E-protein in infected- untreated samples but not in treated samples. Untreated, uninfected samples serve as internal controls (**S5G Fig**). The Ponceau S stained blot showed no proteins upon Proteinase K treatment (**S5G Fig**). Plaque assays further confirmed that exosomes isolated from LGTV-infected N2a cells contain infectious viral RNA, with a significantly higher number of plaques, evident upon infection with exosome fractions in comparison to the infection with supernatant fractions (**Fig 4A and S6A and S6B Fig**). Furthermore, infectious exosomes containing LGTV RNA and proteins prepared from N2a cells at different time points (24, 48, 72 h p.i.) were capable of re-infecting naïve N2a cells (**Fig 4B**). Significantly higher level of viral burden was evident in N2a cells freshly infected with LGTV-containing exosome fractions prepared from 48 or 72 h (p.i.) in comparison to the infection with exosome fractions prepared from 24 h p.i. (**Fig 4B**). Re-infection with supernatant fractions showed undetectable levels of LGTV (**Fig 4B**). Similar levels of viral re-infection kinetics were observed upon incubations with LGTV-infected N2a cell-derived exosomes isolated using commercially available isolation reagent that were used to infect naïve/fresh N2a cells (**S6C Fig**). To find, if mosquito-borne flaviviruses such as WNV viral RNA is also present in exosomes, mouse N2a cells were infected with WNV. Viral infection kinetics showed that WNV readily infected N2a cells with increased viremia at 72 h p.i. (**Fig 4C**). Also, exosomes derived from WNV-infected N2a cells showed a peak in viral burden at 72 h p.i (**Fig 4D**), suggesting that WNV RNA is also present in exosomes.

### Exosome-mediated viral transmission is clathrin dependent

We treated (4 h) N2a cells with 5 μg of 4G2 monoclonal antibody, followed by infection with exosomes from LGTV-infected (72 h p.i.) N2a cells to analyze if treatment with 4G2 antibody affects viral transmission. No differences were found in antibody treated or untreated groups (**Fig 4E**). Next, we determined if exosome mediated viral transmission is receptor-dependent and requires clathrin-mediated endocytosis. We treated N2a cells with clathrin specific inhibitor (Pitstop-2; 30 μM and 15 min), and infected these clathrin-inhibitor treated cells with infectious (LGTV; 72 h p.i.) exosomes derived from N2a cells. We noted significant (P<0.05) reduction in LGTV loads (72 h p.i.) in Pitstop-2 treated cells in comparison to the DMSO (vehicle) treated controls (**Fig 4F**). These results suggest that exosome-mediated LGTV transmission to naïve cells is receptor-dependent endocytosis that requires clathrin.

### Exosome release inhibitor GW4869 reduces LGTV loads and transmission

**Fig 5:**
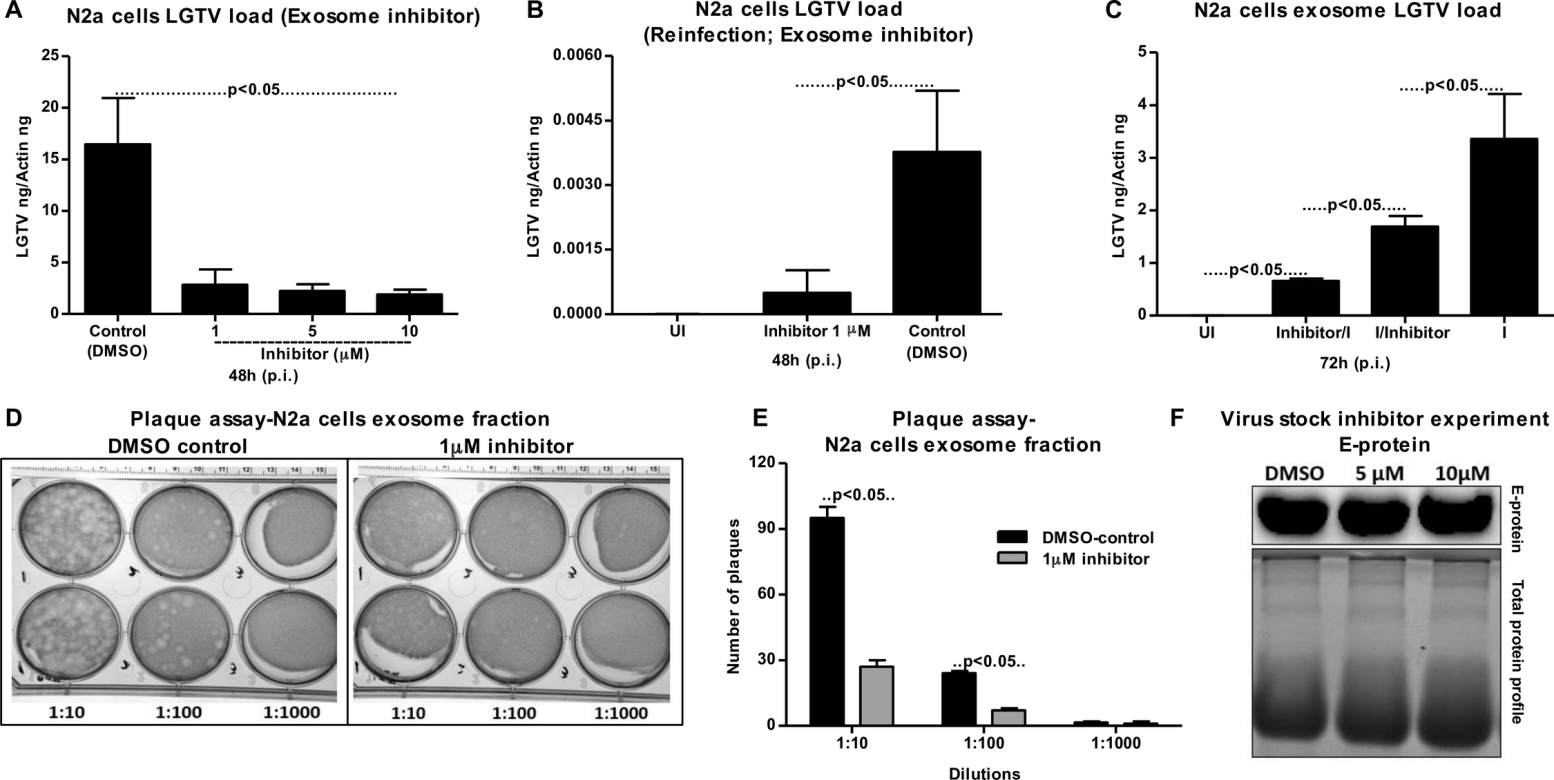
Treatment of neuronal N2a cells with exosome inhibitor affects LGTV infection, if treated either before or after infection. (A) QRT-PCR analysis showing levels of LGTV in exosomes isolated from N2a cells at 48 h p.i. in the presence of exosomes-inhibitor at different concentrations (1, 5, 10 μM). 1 x 10^5^ N2a cells were pre-treated with inhibitor for 4 h followed by infection with LGTV (6 MOI). Exosomes isolated from N2a cells treated with DMSO serve as loading control. (B) Levels of LGTV in N2a cells at 48 h p.i. infected by treatment with exosomes (20 μl) isolated from control or inhibitor-treated N2a cells is shown. UI indicates uninfected cells that serve as control. (C) QRT-PCR analysis showing levels of LGTV in N2a cells at 72 h p.i. Uninfected (UI) N2a cells were treated with 5 μM inhibitor. For LGTV-infection, N2a cells were first treated for 4 h with inhibitor then followed by infection (Inhibitor/I) or cells were first infected for 4 h with LGTV and then treated with inhibitor (I/inhibitor). LGTV (6 MOI) was used to infect 1 x 10^5^ N2a cells in the presence (5 μM) or absence of inhibitor. LGTV transcript levels were normalized to mouse beta-actin. (D) Plaque assays performed with different dilutions (1:10, 1:100, 1:1000) of exosomes fraction isolated from control DMSO-treated or inhibitor-treated N2a cells is shown. A representative image from two independent experiments is shown. (E) Quantitative assessment of number of plaques from DMSO-treated or inhibitor-treated (D) is shown. P value determined by Student’s two-tail *t* test is shown. (F) Laboratory virus stocks treated with either GW4869 inhibitor (5 and 10 μM) or DMSO as control is shown. Representative data in other panels is shown from at least three independent experiments.

Presence of exosome-inhibitor at different concentrations (1, 5 and 10 μM) significantly (P<0.05) reduced LGTV loads in exosomes (from N2a cells) in comparison to DMSO-treated controls (**Fig 5A**). In addition, exosomes prepared from inhibitor-treated (1 μM) N2a cells were significantly reduced in re-infecting naïve N2a cells in comparison to DMSO-treated control group (**Fig 5B**). Furthermore, we found that exosomes isolated from N2a cells pre-treated with 5 μM inhibitor before LGTV infection had significantly lower viral loads in comparison to exosomes isolated from cells post-treated with inhibitor after infection (**Fig 5C**). However, viral loads in exosomes were significantly reduced in N2a cells irrespective of pre- or post- inhibitor treatment in comparison to the infection performed with LGTV from laboratory viral stocks with known titers (5 MOI) (**Fig 5C**). Plaque assays performed with LGTV-infected N2a cell-derived exosomes isolated from DMSO-treated group yielded significantly (P<0.05) increased number of plaques in comparison to the number of plaques with exosomes isolated from inhibitor-treated group (**Fig 5D and 5E**). Also, plaque assays performed with exosome fractions from N2a cells revealed the viral titers for both N2a-DMSO control group (8 x 10^3^ pfu/ml) and N2a 1 μM-inhibitor treated group (2.3 x 10^3^ pfu/ml). We also determined the effects of GW4869 inhibitor on LGTV viral particles from laboratory virus stocks. Immunoblotting with 4G2 antibody showed no differences in 5 or 10 μM treated (4 h) groups, in comparison to the DMSO control (**Fig 5F**). This data suggested that GW4869 has no effect on viral particles.

### Infectious LGTV RNA is transmitted from one neuronal cell to others via exosomes in primary cultures of mouse cortical neurons

**Fig 6:**
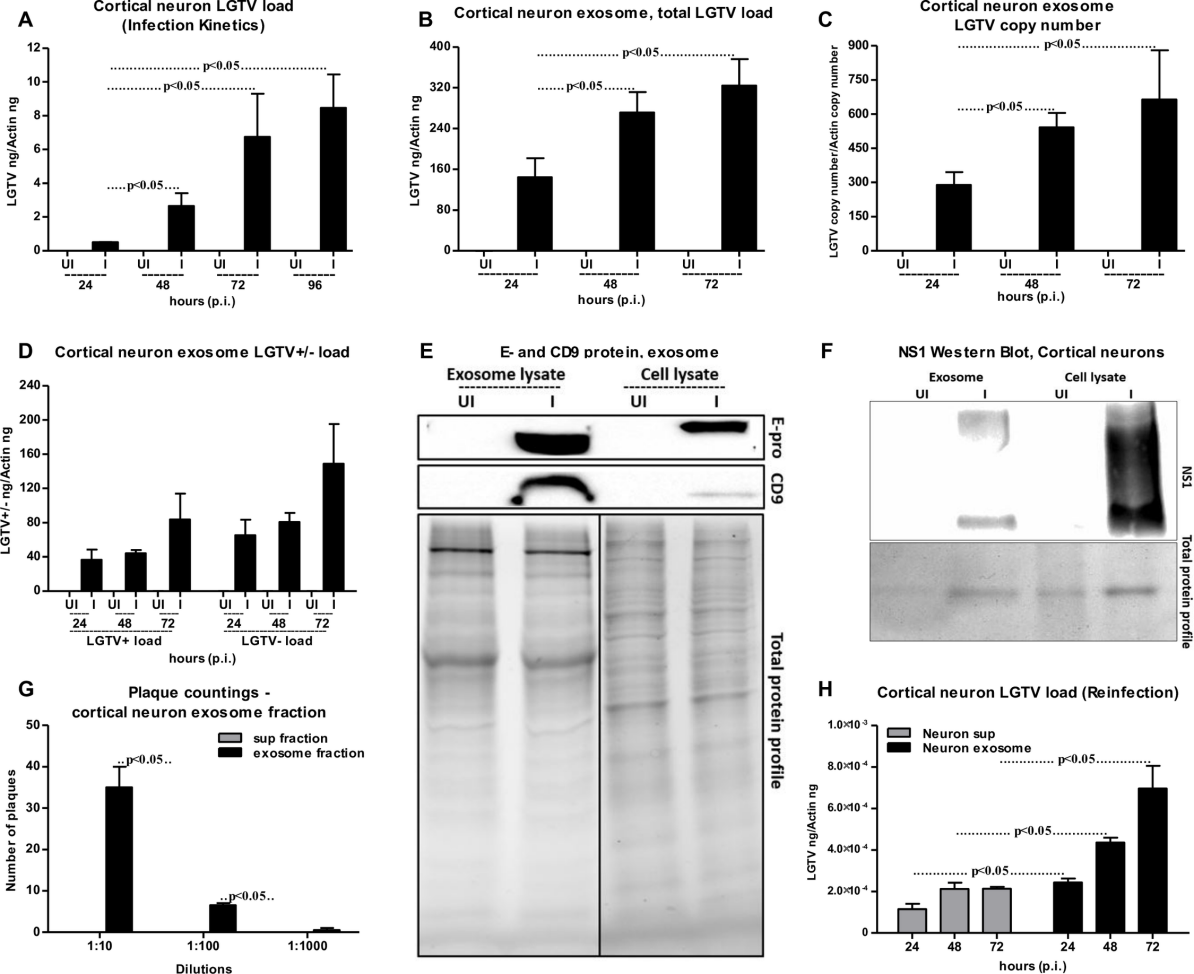
Detection of LGTV RNA and proteins in exosomes isolated from primary cultures of mouse cortical neurons. LGTV (4 MOI) was used to infect 1 x 10^5^ murine cortical neuronal cells. UI indicates uninfected and I indicates LGTV-infected. (A) QRT-PCR analysis showing LGTV infection kinetics in primary cultures of mouse cortical neurons at different time points (24, 48, 72, 96 h p.i.). Total LGTV loads in exosomes isolated from cortical neurons (B), copy numbers (C) and levels of LGTV positive-sense strand or negative-sense strand (D) in exosomes isolated from cortical neuronal cells at different time points (24, 48 and 72 h p.i.). (E) Immunoblotting analysis showing detection of LGTV E-protein and mammalian exosomal marker CD9 in exosome fraction and total lysates from whole cells prepared from uninfected (UI) or infected (I) cortical neuronal cells at 48 h p.i. Stain-free gel showing total protein profile serves as the loading control. (F) Immunoblotting analysis showing NS1 levels in total neuronal lysates and exosomes derived from LGTV-infected (MOI 4; 72 h p.i.) cortical neurons. Uninfected (UI) cells and exosomes derived from these cells serve as controls in addition to total protein profiles. For immunoblotting assays, 2 x 10^7^ cortical neuronal cells were infected with 4 MOI of LGTV. (G) Quantitative assessment of number of plaques from exosomal and supernatant fractions is shown. (H) QRT-PCR analysis of viral loads in 1x 10^5^ naïve cortical neuronal cells at 24 h p.i., infected through treatment with exosomes (20 μl) or supernatant fractions (400 μl) prepared from 24, 48 and 72 h p.i., LGTV-infected neurons show presence of LGTV. LGTV transcript levels were normalized to mouse beta-actin. P value determined by Student’s two-tailed *t* test is shown. Representative data is shown from at least three independent experiments.

**Fig 7:**
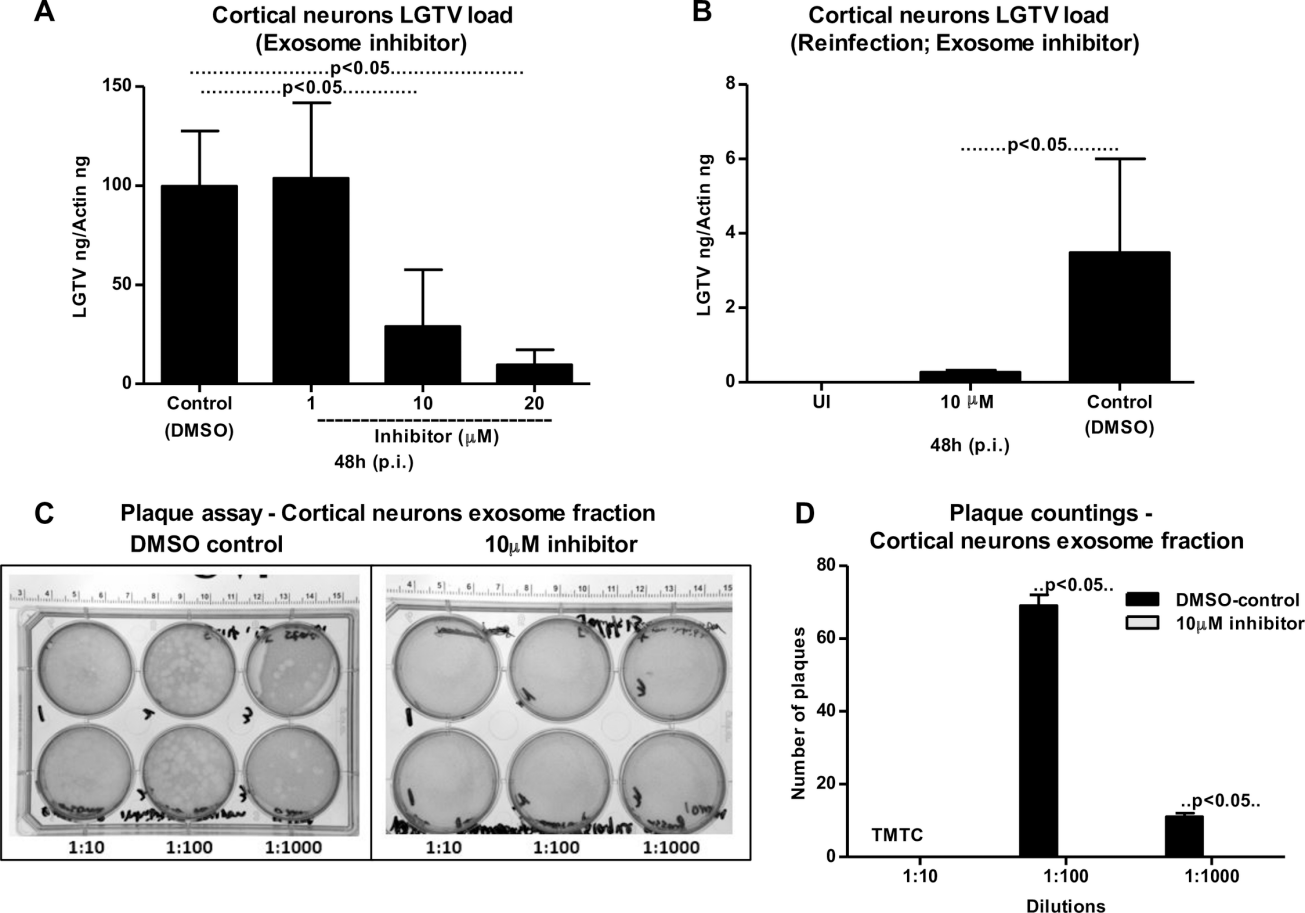
Treatment of primary cultures of cortical neurons with exosome inhibitor affects LGTV infection. (A) 1 x 10^5^ cortical neuronal cells was infected with LGTV (4 MOI). QRT-PCR analysis showing levels of LGTV in exosomes isolated from cortical neuronal cells at 48 h p.i. in the presence of exosome-inhibitor at different concentrations (1, 10, 20 μM). Cortical neuronal cells were pre-treated with exosomes inhibitor for 4 h followed by infection with LGTV. Exosomes isolated from cortical neuronal cells treated with DMSO served as control. (B) Levels of LGTV in fresh cortical neuronal cells at 48 h p.i., infected by treatment with exosomes (20 μl) isolated from control or inhibitor-treated mouse cortical neuronal cells is shown. UI indicates uninfected cells that serve as control. LGTV transcript levels were normalized to mouse beta-actin. (C) Plaque assays performed with different dilutions (1:10, 1:100, 1:1000) of exosomes fractions isolated from control DMSO-treated or inhibitor-treated cortical neuronal cells are shown. Ruler at the top determines the scale for the plaque assay. Representative images are shown from two independent experiments. (D) Quantitative assessment of the number of plaques from (C) is shown. TMTC indicates “too many to count”. P value determined by Student’s t test is shown. Representative data in A and B is shown from three independent experiments.

To analyze whether exosomes are the mediators of viral transmission from one neuronal cell to other in an *in vivo* model, LGTV infections were performed on primary neuronal cultures of murine cortical neurons (isolated from embryonic day E16 brains, as described in methods). Infection of cortical neurons with LGTV (MOI 4) showed time dependent kinetics of LGTV infection with increased viral burden at 72–96 h p.i. (**Fig 6A**). QRT-PCR analysis revealed significantly (P<0.05) increased LGTV total loads and copy numbers in exosomes isolated from murine cortical neurons at 72 h (p.i.) when compared to exosomes isolated from other tested time points (24 and 48 h p.i.) (**Fig 6B and 6C**). We also detected higher loads of LGTV positive- and negative- sense RNA strands, suggesting presence of both viral genomes in the exosomes derived from infected-cortical neurons (**Fig 6D**). Immunoblotting showed abundant LGTV E-protein amounts (2–3 folds) in exosomes isolated from cortical neurons in comparison to the loads found in total cell lysates (**Fig 6E**). Similar to N2a cells, possibly de-glycosylated LGTV E protein (with low molecular mass) was detected in exosomes isolated from cortical neurons in comparison to the total cell lysates (with high molecular mass) prepared from cortical neurons (**Fig 6E**). Elevated levels of CD9 (exosomal enriched marker) were found in the exosomes derived from LGTV-infected cortical neuronal cells and in total cell lysates in comparison to their respective uninfected controls (**Fig 6E**). In addition, levels of CD9 were dramatically elevated in exosomes from LGTV-infected cortical neuronal cells in comparison to the levels in total cell lysates (**Fig 6E**), supporting that LGTV infection may regulate the enrichment of CD9 in neuronal exosomes. Also, exosomes derived from LGTV-infected cortical neurons showed higher amounts of CD9, when compared to the N2a cell-derived exosomes containing LGTV (**Figs 6E and 3J**). Total protein profiles served as loading control in the immunoblotting analysis (**Fig 6E**). Immunoblotting showed presence of NS1 in both exosome fractions and in total cell lysates suggesting that exosomes from cortical neuronal cells contain LGTV proteins (**Fig 6F**). Plaque assays confirmed that exosomes isolated from cortical neurons carry infectious and replicative viral RNA, since significantly increased number of plaques were evident upon infection with exosome fractions (in different dilutions; 1:10, 1:100, 1:1000) in comparison to the infection with supernatant fractions (**Fig 6G and S7A and S7B Fig**). Similar observations were previously noted for N2a cells, suggesting that LGTV is enriched in neuronal exosomes. Additionally, exosome fractions prepared from LGTV-infected cortical neurons at different time points (24, 48, 72 h p.i.) were capable of re-infecting naïve primary cultures of cortical neurons (**Fig 6H**). A significant higher viral burden was evident in the cortical neurons infected with exosome fractions (prepared from 24, 48, 72 h p.i.) in comparison to the infection with the supernatant fractions prepared from respective time points (**Fig 6H**). These data suggest that exosomes derived from LGTV-infected neuronal cells are potential mediators for spreading infection to other neurons. Furthermore, presence of exosome-inhibitor at concentrations of 10 or 20 μM significantly (P<0.05) reduced viral infection in cortical neurons in comparison to DMSO-treated controls (**Fig 7A**). However, no differences in the viral burden of cortical neurons were noted upon treatment with 1 μM exosome inhibitor in comparison to DMSO-treated control (**Fig 7A**). This data suggested that neuronal cells in *in vivo* might produce higher number of exosomes that could not be inhibited with less concentration (1 μM) of inhibitor. Exosomes isolated from 10 μM-treated cortical neurons showed significantly (P<0.05) reduced re-infection of naïve cortical neurons in comparison to the infections performed with exosomes isolated from DMSO-treated control group (**Fig 7B**). Plaque assays confirmed that LGTV-containing exosomes isolated from DMSO-treated neurons contained viable and increased LGTV loads in comparison to exosomes isolated from 10 μM exosome inhibitor-treated group (**Fig 7C and 7D**). Collectively, these results suggest that LGTV and perhaps TBEV, uses exosomes as novel modes of transmission from one neuronal cell to the other.

## Discussion

Exosomes contribute to the transmission of intracellular information from one cell to another and from one tissue to the other [2,30,61]. Several biological implications and medical applications have been associated with the exosomes as potential mediators of communication between cells and tissues [3,20,62,63]. For the first time our study shows that exosomes are novel mediators for transmission of arthropod-borne flaviviruses that infect a wide variety of vertebrate hosts including humans. Our discovery that tick cells secrete exosomes and that these exosomes are the carriers of tick-borne LGTV (**Fig 1**) suggest that other tick-borne flaviviruses such as TBEV and POWV might also use this novel mode of transmission from arthropods. Cryo-EM data showed that arthropod or neuronal cell-derived exosomes are of variable sizes and were in the ranges of 30–250 nm (**Figs 1A and 3A**). Exosomes isolated from both arthropod and neuronal cells had majority of the exosome sizes between 50–100 nm and fewer vesicles from sizes of 200–250 nm in both uninfected and infected groups (**Figs 1B, 1C, 3B and 3C**), suggesting purity in isolation methods. Increased number of exosomes in LGTV-infected in comparison to the uninfected groups (**Figs 1D and 3D**), suggested higher production and release of exosomes. Our immediate future avenue determines the loads and activity of the neutral sphingomyelinase in LGTV-infected arthropod and neuronal cells. To make virus preparations for structural studies, concentrated supernatants or titers with 10^9^ to 10^12^ PFU/ml and centrifugal forces of 200,000g are used [64], that are not similar in exosomal preparation methods. However, in order to minimize the viruses and large protein aggregates that co-sediments during ultracentrifugation, we adopted the buoyant density of exosomes for purification purposes. Continuous or discontinuous sucrose density gradient centrifugation has been used extensively to purify exosomes. However, this method does not allow separation of exosomes from viruses and macro vesicles or large microvesicles with comparable sedimentation velocities [55]. Substituting sucrose with iodoxanol (OptiPrep) in the velocity gradients using 5–40% density gradients has been shown to overcome the limitations and result in purified exosomal preparations [55]. Detection of tick HSP70 in exosomal fractions (**Fig 1H**), suggested it to be a novel arthropod marker that may be present in exosomes from saliva and facilitate tick feeding on vertebrate host. Our recent study reported that arthropod HSP70 may aid in the host fibrinogenolysis at the tick bite site [65]. Detection of CD9 in all uninfected fractions and enrichment in fractions four and five suggested these fractions to be exosomes. The observed shift in enrichment of CD9 in LGTV-infected fraction three and four and no detection in fractions one and six suggested presence and enrichment of other proteins or cargo (including viral E-protein in fraction four) in those fractions. Our findings showing the enrichment of both arthropod and neuronal E-protein in exosomal fractions four and five confirmed the presence of viral E-protein in exosomes (**Figs 1E and 3E**). We hypothesize that due to space limitation and tightly regulated cargo sorting mechanisms, exosomes are certainly filled with viral RNA and proteins that are trafficked to extracellular space and later recycled back through fusion with plasma membranes. If virions or entire viral particle are perhaps exported through exosomes, we could anticipate enclosure (or packaging) of few LGTV viruses of 40–60 nm size in ~150–200 nm diameter of arthropod/neuronal cell-derived exosomes. We did not detect any viral particles or fully assembled virions inside of the exosomes, in several of our preparations processed for cryo-electron microscopy. However, we do not exclude the possibility of viral particles presence in the exosomes. Based on our findings, we believe that if viral RNA (both positive and negative strands) and proteins are loaded into exosomes, they can be exported and subsequently transmitted to the neighboring cells and distant tissues for pathogenesis in short times. The matured virions containing positive sense RNA strand exit cells through membrane budding. On the other hand, the replicative viral RNA genome will have a negative RNA strand and are cytosolic [17,54,66–68]. Detection of both positive and negative-sense RNA strands in tick/neuronal cell-derived exosomes suggest that exosomes facilitate transmission of both negative and positive-strand RNA genomes. The higher loads of negative-strand RNA in the exosomes derived from neuronal cells implied that LGTV negative strand RNA may simply get trafficked during endocytosis/uptake by these cells. The negative-strand of RNA generally exists as dsRNA with positive-strand. Thus, it seems that dsRNA may be present inside exosomes rather than single-stranded positive or negative strand of LGTV. In addition, entry of more viral RNA and proteins inside cells via receptor-mediated endocytosis may simply force the replicative viral RNA to exit the host cell and seek other neighboring cells through exosome-mediated transmission. Our finding that exosome mediated viral transmission is dependent on clathrin (**Fig 4F**) further suggest an important role for exosomes as viral RNA and protein transporters.

Up-regulation of some proteins in LGTV-infected tick exosomal lysates in comparison to the uninfected controls suggests the importance of these proteins in facilitating the transmission of tick-borne flaviviruses from tick cell-derived exosomes (**Fig 1H**). Our current efforts are focused in identification and characterization of these important cargo proteins on arthropod exosomes that could be candidates for the development of novel transmission-blocking vaccine(s) [69]. The presence of LGTV RNA (as determined by RNase A treatment studies) and E- protein inside exosomes but not outside in suspensions of exosomal fractions, suggest that exosomes not only securely carry the viral RNA (both positive and negative strands), but also transport the essential viral E-protein into host endosomal membranes for release of viral content inside cells. Our finding that exosome mediated viral transmission is clathrin-dependent suggests a possible receptor-mediated endocytosis uptake of infectious exosomes into naïve cells. Our transwell assays with tick cell-derived exosomes and human keratinocytes (**Fig 1M**) suggest that tick spit/secreted saliva (that could contain exosomes loaded with LGTV viral RNA and proteins) could facilitate transmission of this virus from the bite site to the vertebrate host skin cells. No differences in time course of LGTV infection in human keratinocytes suggested that these cells may not keep persistent infection, but may transmit viruses to dendritic/other migratory immune cells in the skin at their earliest. We also assume that keratinocytes are probably highly immune tolerant and may maintain viral infections to lower peaks. Abundance of LGTV infectious RNA and proteins in exosomes also suggests that exosomes may readily facilitate the dissemination of these viral factors within the tick body (for example, from midgut, upon entry, and through hemolymph to salivary glands during transmission) or transmission through saliva to the vertebrate host upon infected arthropod bite or blood feeding. Infection of vascular endothelial cells with tick cell-derived exosomes containing LGTV infectious RNA and proteins suggests that upon tick blood feeding, arthropod exosomes facilitate infection of the blood endothelium in vertebrate host. It is noteworthy that GW4869 inhibitor significantly (P<0.05) lowered LGTV in exosomes derived from tick, bEnd.3, N2a and neuronal cells (**Figs 1M, 2D, 5A and 7A**). These data suggest a common pathway shared in the production and release of exosomes in both arthropod and vertebrates. Overall, these studies revealed a novel mode of flavivirus transmission from the arthropod vector to the vertebrate host via arthropod exosomes that could be envisioned as transmission-blocking strategies.

Most of the flaviviruses can infect and replicate in the vertebrate brain microvascular endothelial cells that line and guard the BBB. Infected endothelial cells allow these flaviviruses to enter and cause neuroinvasion of the CNS [70–73]. We hypothesize that initial entry of few infected exosomes derived from endothelial cells, lining the BBB may lead to virus transmission into the CNS. Infection of neuronal cells and secretion of abundant loads of infectious exosomes by neuronal cells may promote the breaching of the BBB, thereby allowing entry of higher peripheral viral loads, in addition to trafficking of immune cells from the periphery. Based on our results (**Fig 2**), we assume that initial batch of infected-brain microvascular endothelial (bEnd.3) cell-derived exosomes containing higher loads of infectious LGTV RNA and proteins may enter into the CNS at an early time point (24 h p.i. of endothelial cells). The higher viral loads in brain endothelial cell-derived exosomes from early time points (24 h p.i.) in comparison to lower loads in exosomes at later time points further suggest earlier transmission of viral RNA through exosomes that infects neighboring neuronal cells. We also noted that bEnd.3 cells (in infection kinetics assays) were more resistant to LGTV infection with no severe cytopathological effects when compared to neuronal cells. We hypothesize that the brain endothelial cells may not support the higher rate of viral replication or persistent infection for longer times. This could result in higher packaging of LGTV viral RNA and perhaps proteins in bEnd.3 cell-derived exosomes that would lead to dissemination of flaviviruses to neuronal cells at earlier times. The transwell assay data (**Fig 2D**) mimic *in vivo* scenario, where exosomes derived from infected-bEnd.3 cells might transmigrate through astrocyte foot layer and infect neurons in the CNS. This data could be directly related to the *in vivo* situation that proposes virus transmission from infected-brain microvascular endothelial cells (lining the BBB) to the interior of the CNS. Taken together, these studies imply that infected-brain endothelial cells may not entertain flavivirus replication for longer times and hence transmit these viral RNA and proteins to their neuronal counterparts at the earliest and via exosomes.

Our study also suggest that exosomes derived from neuronal cells likely able to mediate transmission of tick-borne flavivirus RNA and proteins from one neuronal cell to the other in the CNS. Higher loads of E-protein (2–4 folds more) in exosomes derived from murine cortical neurons in comparison to the *in vitro* cultures of N2a cell-derived exosomes suggest higher packaging of viral RNA and proteins in cortical neurons (**Fig 6E**). We believe that the observed lower mass for LGTV E-protein in both N2a cells and cortical neurons is due to possible de-glycosylation of the E- protein. The de-glycosylated E-protein in laboratory viral stocks suggested mixture of virions with exosomes in those frozen supernatants. However, this effect was not evident in arthropod cell-derived exosomes, suggesting that E-protein in arthropod exosomes may not undergo protein modification. Glycosylated form of LGTV E- protein in arthropod cells is maintained possibly to facilitate exosome fusion and viral infection of host cells immediately upon host seeking and tick blood feeding on vertebrate host. It has been also observed that in mosquitoes, WNV E-protein is heavily glycosylated and is required for pathogen transmission to the vertebrate host [74]. We assume that presence of de-glycosylated form of viral E-protein in neuronal and other mammalian cell-derived exosomes may allow viral E-protein to maintain its stability in these small vesicles during transmission from one cell to other. Alternatively, we also hypothesize that higher packaging of viral E-protein in vertebrate neuronal exosomes may be feasible only if E-protein exist in de-glycosylated form with less molecular mass in comparison to the glycosylated form. Also, arthropod and vertebrate host may require different conformations of E-protein that may aid when contents from exosomes are delivered to the host cytosol. The de-glycosylation of E protein in neuronal exosomes may also facilitate higher infectious ability to form matured virions in new hostile environment. Higher loads of CD9 in exosomes derived from neurons suggest that cortical neuronal cells might have greater production of exosomes in comparison to the *in vitro* cultured N2a cell line (**Figs 3J and 6E**). It is reasonable to consider that neurons have complex ways of cell communication such as synaptic transmission and neurotransmitter release that might require greater production of exosomes in the CNS. Low total protein content observed in the N2a and cortical neuronal exosomes compared to the protein content in the whole cells also suggest that only few essential proteins are imported as cargo in LGTV-infected neuronal cell-derived exosomes. Total protein profiles in N2a cells showed absence of some exosomal proteins upon LGTV infection, implying that these proteins may affect or inhibit viral proteins in N2a cell-derived exosomes. Our future studies in identifying these reduced exosomal proteins upon LGTV infection would assist in identifying novel therapeutic targets against transmission. The detection of E-protein at higher molecular mass (<250kDa) in native-PAGE gels, suggested that exosomes might contain higher order structures of E-protein as oligomers. The presence of NS1 in the same samples at similar molecular mass further indicated that exosomal fractions might contain polyprotein (**Fig 3K**). Presence of highly infectious LGTV RNA and proteins in exosomes from neuronal cells suggests that these cells upon infection, mediate dissemination in the CNS. Also, detection of WNV in neuronal cell-derived exosomes, further suggest exosomes as novel transmission modes for both tick- and mosquito-borne flaviviruses in neuronal cells. We assume that exosomes may maintain viability of these viral RNA and proteins that may favor persistent pathogenesis.

**Fig 8:**
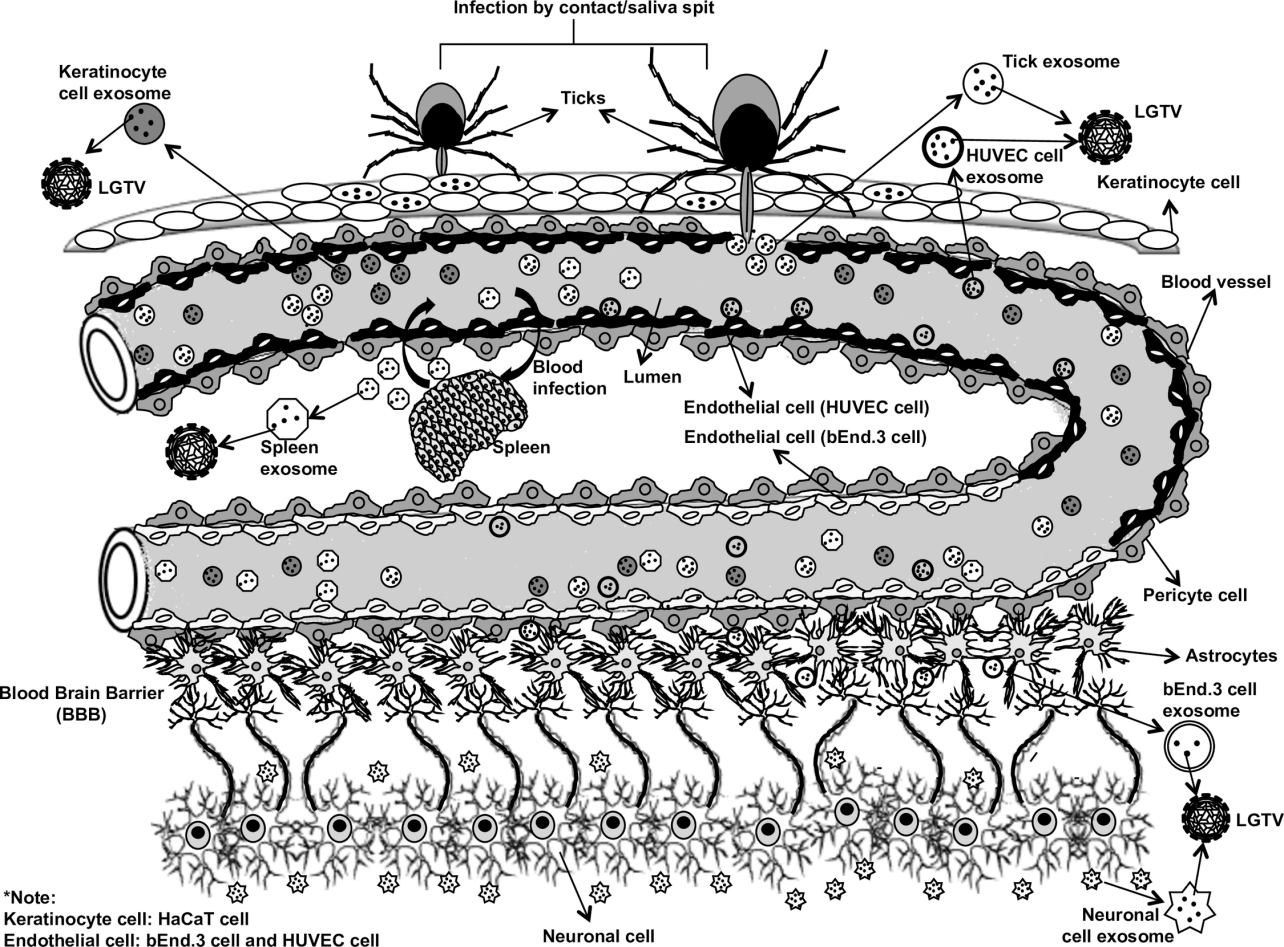
Proposed model showing arthropod-borne flaviviruses transmission to human host via arthropod exosomes. Tick bite or injection/spit of saliva from infected ticks deposits exosomes containing viral infectious RNA or proteins securely to humans. Exosomes derived from tick cells/saliva infect human skin (keratinocytes) or may directly deposit saliva enriched with exosome containing tick-borne viruses into the blood capillaries/vessels. Exosomes derived from vascular endothelial cells containing flaviviruses infect neighboring endothelial cells leading into infection of the peripheral system. Higher viral loads in peripheral tissues increase viremia in blood and eventually allow entry and replication of these arthropod-borne viruses in brain microvascular endothelial cells that line the BBB. Early production and higher loads of flaviviruses in brain endothelial cells would allow these viruses to cross BBB and infect neurons. Infected neurons produce high numbers of exosomes containing infectious RNA and proteins that fuse with cell membranes of naïve neuronal cells thereby infecting neighboring neurons and leading to spread of infection and severe neuronal loss.

GW4869 (dihydrochloride hydrate) is a cell permeable but selective inhibitor for neutral sphingomyelinase (an important enzyme required for the exosome production and release). Effect of this inhibitor on both arthropod and mammalian cells used in this study suggests, presence of neutral sphingomyelinase in these cells. Treatment with GW4869 affected LGTV- replication, loads and transmigration from one cell type to other suggesting that LGTV or other tick-borne flaviviruses may use neutral sphingomyelinase or its related pathway(s) for packaging into exosomes. Future studies would unravel the role of neutral sphingomyelinase on packaging of LGTV and other flaviviruses in arthropod or mammalian exosomes. In N2a cells, 1 μM of inhibitor was sufficient to inhibit the loads of LGTV (as revealed by infection, reinfection and plaque formation) in contrast to higher doses (10 and 20 μM) of GW4869 that was required for inhibition of viral loads in primary cortical neuronal cells (**Fig 7A**). Higher sensitivity of N2a cells to GW4869 could be explained by the possibility of low number of exosomes or less neutral sphingomyelinase in these cells in comparison to cortical neurons. The effects of GW4869 on LGTV infection in N2a cells implied that inhibition of exosomes either before or after infection would affect LGTV loads and transmission (**Fig 5C**). Our data suggested that inhibition of exosomes reduced LGTV loads in both arthropod and mammalian cells and that infection with tick-borne flaviviruses was affected when exosome production and release was hampered with GW4869 treatment. No effects of GW4869 on laboratory viral stocks suggested that it is specific for blocking release of exosomes and has no direct effect on viral particles. It would be interesting to determine whether GW4869 or other novel exosome inhibitor(s) could serve as potential therapeutic approaches for treating flaviviral infections. The proposed model (**Fig 8**) summarizes the role of exosomes in transmission of tick-borne flavivirus RNA and proteins from the arthropod vector to human cells and dissemination of these infectious exosomes within the vertebrate host. Taken together, our study suggests that exosomes play following important roles: 1) In the transmission of tick/mosquito-borne flaviviruses from infected arthropod vector to the vertebrate host cells, 2) In the infection of the human skin keratinocytes and vascular endothelial cells during tick bite/blood feeding, 3) In mediating the infection of brain microvascular endothelial cells (lining the BBB) and crossing these infectious exosomes to allow neuroinvasion and 4) In the infection of neuronal cells resulting in high production of exosomes containing infectious viral RNA and proteins, necessary for the dissemination and infection of naïve neuronal cells in the CNS that leads to neuropathogenesis and severe neuronal loss.

## Materials and methods

### Cell culture and infection of *in vitro* cell lines

*Ixodes scapularis* ISE6 tick cell line was obtained from Dr. Ulrike Munderloh, University of Minnesota. ISE6 cells were grown as per the culture methods provided by Dr. Munderloh [75]. Human keratinocytes (HaCaT cells) or Human Umbilical Vein Endothelial Cells (HUVEC) were obtained from Drs. Loree Heller and John Catravas laboratories, respectively. Vero (African Green Monkey kidney), mouse brain endothelial (bEnd.3 cells) and mouse neuroblastoma Neuro-2a or N2a cells were purchased from ATCC and were grown according to Company guidelines. Briefly, HaCaT, Vero, bEnd.3 and N2a cells were grown in complete DMEM medium containing 5–10% heat-inactivated FBS (Invitrogen/ ThermoScientific). HUVEC cells were grown in human lung MVEC medium (M199 medium containing 150 mg ECGF- bovine brain extract and 20% FBS) kindly provided by Dr. Catravas laboratory. To determine infection kinetics, 1 x 10^5^ cells were seeded in a 12-well plate, infected with various multiplication of infections (MOI 1; tick cells), (MOI 6: Vero, HaCaT, HUVEC, bEnd.3 and N2a cells) of LGTV. Wild type LGTV (LGT-TP21) strain used in this study was obtained from Dr. Alexander G. Pletnev, NIAID, NIH. Cells were collected at different time points (24, 48, and 72 or 96 and 120 h post infection, p.i.) and processed for RNA or protein extractions. Details for infection studies corresponding to the data shown in different figures is mentioned in their respective Figure legends. Briefly, for infection experiments (or re-infection studies) with exosome fractions containing infectious LGTV RNA and proteins, we infected 1 x 10^5^ HaCaT/HUVEC cells or N2a cells with 20 μl (from 150 μl) of tick (6.6 x 10^4^ pfu/ml) or bEnd.3/N2a cells (3.5 x 10^3^ pfu/ml) derived exosomal fractions, respectively. We used same ratio of supernatant fractions (collected from the step before PBS wash during exosome isolation) from tick or bEnd.3/N2a cells. Titers were determined after plaque countings and calculations. For studies with exosomes and exosome-free supernatant fractions, infected cells (infected with exosomes or supernatant fractions collected at different time points) were either collected at 24 or 48 or 72 h p.i. and processed for RNA extractions. For infection of mouse N2a cells with WNV, we used CT2741 wild-type strain (MOI 5) and analyzed cells for WNV loads in cells and exosomes at different time points (24, 48 and 72 h p.i.).

### Cryo-electron microscopy

Exosomes were vitrified as previously described [76,77] on carbon holey film grids (R2x2 Quantifoil; Micro Tools GmbH, Jena, Germany; or C-flat, Protochips, Raleigh, North Carolina). Briefly, purified concentrated suspensions of exosomes in PBS were applied to the holey films in a volume of ca. 3 μl, blotted with filter paper, and plunged into liquid ethane cooled in a liquid nitrogen bath. We used computerized Vitrobot plunger (FEI, Hillsboro, OR) for freezing. Frozen grids were stored under liquid Nitrogen and transferred to a cryo-specimen holder (70 deg. 626, Gatan, Inc., Pleasanton, CA, or 2550 cryo-tomography holder, E.A. Fischione Instruments, Inc., Export, PA) under liquid Nitrogen before loading into a JEOL 2200FS, or a JEOL 2100 electron microscopes (JEOL Ltd., 3-1-2 Musashino, Akishima, Tokyo 196–8558, Japan). JEOL 2200FS was equipped with in-column energy filter (omega type) and a field emission gun (FEG); JEOL 2100 had a LaB_6_ filament, both were operating at 200 keV. Grids were maintained at near-liquid Nitrogen temperature (-172–-180°C) during imaging. Preliminary screening and imaging of exosomes was done using a 4k x 4k Gatan US4000 CCD camera (Gatan, Inc., Pleasanton, CA), and final imaging was done at indicated 40,000x magnification with a 5k x 4k Direct Electron Detector camera (DE-20, Direct Electron, Inc., San Diego, CA) using a low-dose imaging procedure. An in-column omega electron energy filter was used during imaging with a zero-loss electron energy peak selected with a 20 eV slit. Images were acquired with a ca. 20 electrons/Å^2^ dose; the pixel size corresponded to 1.5 Å on the specimen scale. We used a 2.0–2.3 μm defocus range for imaging. Overall, individual exosome images were acquired from two-three independent batches of exosomes from tick and N2a cells. For quantitation of exosomes size ranges, we manually analyzed the sizes using scale bar from cryo-EM images and counted exosomes per image in each group. Three independent estimations and countings were performed without any bias. Percentages (for size determination) were calculated based on the total number of exosomes in each size range. In addition, total number of exosomes/cryo-EM images were counted and analyzed.

### OptiPrep density gradient exosome (DG-Exo) isolation from concentrated culture medium

Tick cells (1.2 x 10^7^ cells cultured in 12 of Nunc tubes; ThermoScientific) or N2a neuronal cells (8 x 10^7^ cells cultured in 8 different T75 flask; Greiner) were infected with either 1 MOI (tick cells; six of each tube) or 5 MOI (five of each flask with N2a cells) of LGTV. Remaining tubes (6) or flasks (4) were maintained as uninfected controls. The detailed protocol is shown as **S1C Fig**. Supernatants (20-50ml) from uninfected/infected cells of respective cell type were collected and centrifuged at 4°C (480g for 10 min followed by 2000g for 10min to remove cell debris and dead cells). Cell culture supernatants were either first filtered (using 0.1 μm filtering devices; VACUCAP filter for conical tubes; Pall Laboratory/VWR) and concentrated to 2–2.5ml using the Corning Spin-X UF concentrators or centrifugal filter device with a 5 k nominal molecular weight limit (NMWL). The concentrated culture medium were processed for OptiPrep (DG-Exos) isolation as described [55]. In case of OptiPrep (DG-iso) on laboratory virus stocks (7.4 x 10^6^ pfu/ml), we added concentrated stocks of 1.5 ml supernatants directly on the gradient cushion. Briefly, discontinuous gradient of 40% (w/v), 20% (w/v), 10% (w/v) and 5% (w/v) solutions of iodixanol was prepared from the stock solution of OptiPrep 60% (w/v) of aqueous iodixanol (Axis-Shield PoC, Norway) with 0.25M Sucrose/10mM Tris, pH 7.5. We used the polycarbonate bottles with cap (Beckman Coulter) and maximum volume capacity of 26.3 ml to load the discontinuous gradient of iodixanol (4ml each of 40% (w/v), 20% (w/v), 10% (w/v) and 3 ml of 5% (w/v) from bottom to top). The cell culture supernatants (2–2.5ml) was overlaid onto the top of the gradient, and centrifuged at 100,000g for 18 h at 4°C. Six individual uninfected or infected fractions of ~3ml were collected (from top to bottom) manually (with increasing density) and diluted with 5ml of sterile PBS. Fractions were centrifuged at 100,000g for 3 h at 4°C, and followed by one more wash with 5ml of PBS and resuspended in 80 μl PBS. DG-Exos were stored in -80°C and used for analysis.

### Isolation of exosomes from cell culture supernatants

Exosomes were isolated and purified by either DG-Exo gradient method as described before or differential ultracentrifugation method as described by [29]. Details for exosome isolation procedure and modifications (used in this study) are also schematically shown and discussed in **S1 and S2 Figs** and in corresponding figure legends. Briefly, cells were seeded for exosomal- RNA (5 x 10^6^ tick cells; 1 x 10^5^ of either bEnd3.1 or N2a or murine cortical neurons) or protein (1 x 10^6^ tick cells, 2 x 10^6^ N2a cells or 2 x 10^7^ cortical neurons) extractions in either 12/6-well or 10cm^2^ plates in complete L15, DMEM or Neurobasal medium with FBS for overnight, respectively. Next day, cells were changed to respective medium containing bovine exosome-free FBS (Systems Biosciences Inc; SBI). Tick cells plated in commercially available exosome-free FBS medium showed severe loss of cells but infectious loads were not affected. After 4–6 hour of medium replacement, cells were infected with LGTV (tick cells MOI 1; bEnd3.1 and N2a cells MOI 6; and cortical neurons MOI 4). Tick cells were susceptible to 2 or 3 MOI of infection and showed massive death, hence we used 1 MOI dose for tick cell infection studies. Cell culture supernatants were spun at 300 x g, for 10 min, cell pellet was discarded and the supernatant was spun again at 2000 x g for 10min. The pellet containing dead cells was discarded and the supernatant was spun again at 10,000 x g for 30 min to remove cell debris. Increased centrifugation times and rotor types is shown to improve exosome yield and purity [57] and, hence we used these modifications for isolation of exosomes from tick cells. Either supernatants were spun at 100,000 x g, for 70 min (for bEnd.3, N2a and cortical neurons) or for 155 min (for ISE6 tick cells). Supernatants collected after this spin step served as supernatant fractions and were used as controls in our study (indicated with * in **S2 Fig**). For plaque assays performed in this study 600, 60 and 6 μl and for infection studies 400 μl of supernatant fractions were used for all except HUVEC cells (300 μl). The pellets containing exosomes and any contaminants were washed one- time with ice-cold PBS and spun again at 100,000 x g, for either 70 min (for bEnd.3, N2a, cortical neuronal cells) or 155 min (for tick cells), respectively. Resulting exosomes pellet is referred as exosome fractions in this study. Freshly prepared exosome pellets were collected in PBS (and stored frozen at -80°C for re-infection studies performed on uninfected cells or for plaque assays or other tested evaluations) or resuspended in RNA lysis buffer for total RNA extractions, or in modified RIPA buffer (G-Biosciences, BioExpress) for total protein extractions. We also isolated exosomes from N2a cells using the total exosome isolation reagent and extracted total RNA and proteins using total exosome RNA and Protein Isolation kit (Invitrogen/ThermoScientific) as per the manufacturer’s instruction.

### RNA extraction, cDNA synthesis and QRT-PCR analysis

Total RNA from ISE6 tick cells, HaCaT, Vero, HUVEC, bEnd.3, N2a cells or murine cortical neurons infected with various MOI of LGTV or WNV or uninfected controls were extracted using Aurum Total RNA Mini kit (BioRad) following manufacturer’s instruction. Using BioRad iScript cDNA synthesis kit, 1 μg RNA was converted to cDNA and the generated cDNA was used as template for the amplification and determination of the viral burden. For determination of positive- or negative-sense strands of LGTV, we used the iTaq Universal SYBR Green One-Step kit (BioRad) and followed manufacturer’s instructions. For detection of positive- and negative-sense strands of LGTV RNA, we used published forward and reverse primers for Langat prM-E [78]. For WNV detection, published primers for *E* gene were used [73]. To normalize the amount of templates, either tick or mouse or human *beta actin* amplicons were quantified with published primers [73,79]. Equal amounts of tick/mouse/human cDNA samples were used in parallel for beta actin and Langat prM-E. The ratio of Langat prM-E gene copy/beta actin gene copy was used as an index to determine the rate of infection in each analyzed sample. QRT-PCR was performed using iQ-SYBR Green Supermix (BioRad, USA). Standard curves were prepared using 10-fold serial dilutions starting from standard 1 to 6 of known quantities of *actin* or Langat *prM-E* gene fragments and QRT-PCR reactions were performed as described [72,73,79]. To determine the copy number of viral RNA in exosomes, we used the LGTV RNA values with standards and converted to copy numbers using the formula: Number of copies (molecules) = (amount of amplicon) ng x 10^23^ molecules per mole/(length of dsDNA amplicon * 660g per mole)† *1 x 10^9^ ng per g. Alternatively, we also used the online calculator to convert to copy numbers (http://scienceprimer.com/copy-number-calculator-for-realtime-pcr). For RNase A treatment, we isolated fresh exosomes from either uninfected or LGTV-infected N2a cells (2 x 10^7^), distributed the infected exosomes as treated (5 μg/ml RNase, 37°C for 15 min) or untreated groups. Exosomes were also treated with Triton X-100 (0.1%, for 45 min at RT) and then followed by treatment with RNaseA as before. N2a cells (2 x 10^5^) were infected (72 h p.i.) with these treated or untreated LGTV-infected exosomal samples were processed for RNA extractions and QRT-PCR. Untreated exosomal samples from uninfected group served as internal controls.

### Immunoblotting

Western blotting was performed as described [72,73]. For DG-Exos samples, equal volume (20 μl) of each fraction from 1–6 or 20 μg of total protein lysates from uninfected and infected cells or 10 μl of each fraction from virus stock samples were loaded onto 12% SDS-PAGE, followed by immunoblotting and labeling with highly cross-reactive 4G2 monoclonal antibody to detect LGTV E-protein or exosomal specific markers such as HSP70 (rabbit polyclonal; Cell Signaling Technologies, Inc) or CD9 (mouse monoclonal; Invitrogen/ThermoScientific) and respective secondary antibodies (Santa Cruz Biotechnologies, Inc). For immunoblotting using cell lysate and exosome lysates, briefly, 5 x 10^6^ ISE6 tick cells, or 2 x 10^6^ N2a cells or 2 x 10^7^ cortical neurons were seeded in 10 cm^2^ plates and allowed to settle/adhere for overnight. Next day, we changed the media on N2a cells and neurons to DMEM or Neurobasal medium, respectively containing bovine exosome free FBS (Systems BioSciences, Inc; SBI). ISE6 cells were retained with complete L-15 media containing 5% regular FBS (to avoid massive cell death and loss observed when processed for exosome isolation using commercially available exosome-free FBS; SBI). After 4–6 hours of media replacement, cells were infected with LGTV (tick cells, MOI 1; N2a cells, MOI 6 and cortical neurons, MOI 4). After 72 h (tick cells) or 24, 48, 72 h p.i. (N2a cells and neurons), cell culture supernatants were collected and processed for exosome isolation by ultracentrifugation (See **S2 Fig)**. The exosome fractions collected after PBS wash and the adherent cells collected from same plates (washed twice with 1 x PBS), were resuspended in modified RIPA buffer. Total protein amounts were estimated using BCA kit (Pierce/ThermoScientific). We loaded 25 μg (tick cells) or 30–35 μg (N2a and cortical neurons) of total cell lysates or total exosomal proteins and separated them on either 12% (Laboratory casted) or precasted 4–20% SDS-PAGE gradient stain-free gels (NuSep; BioExpress). Followed by gel electrophoresis, blots were blocked in buffers and probed with either highly cross-reactive 4G2 (obtained from Dr. Michel Ledizet, L2 Diagnostics; under non-reducing conditions) or CD9 (Invitrogen/ThermoScientific; under non-reducing conditions) or monoclonal anti-Langat virus NS1 (Clone 6E11; BEI Resources) antibodies, followed by mouse monoclonal HRP-conjugated secondary antibodies (Santa Cruz Technologies, Inc). Total protein profiles (images obtained from stain free gels after running or imaged from Coomassie stained gels) serve as loading controls. For protease-resistance assay using proteinase K (that generally digest proteins in biological samples), we used typical working concentrations of 50–100 μg/ml (for tick cells; 50 μg/ml) or much above the concentrations (for N2a cells; 100 μg/μl). Briefly, we isolated fresh exosomes (ultracentrifugation methods) from tick (2 x 10^6^) or N2a cells (2 x 10^7^), and treated with Proteinase K for 15 min at 37°C. Samples were then heat-inactivated at 60°C for 10 min and loaded on SDS-PAGE gels and processed for immunoblotting with 4G2 antibody followed by relevant secondary antibody. Antibody binding was detected with WesternBright ECL kit (Advansta, BioExpress). Blots were imaged using Chemidoc MP imaging system and processed using Image Lab software from the manufacturer (BioRad).

### Native-PAGE analysis

For the native-PAGE analysis, we seeded ISE6 tick cells (2 x 10^6^) in regular L15 medium for overnight and infected with LGTV (MOI 1). For N2a cells (5 x 10^6^), we plated them in regular complete DMEM medium and allowed them to adhere for overnight, cells were then replaced with exosome-free FBS medium. After 4 h of media change, N2a cells were infected with LGTV (6 MOI). Post 72 h of infection, tick/N2a cell culture supernatants were processed for isolation of exosomes. Exosomes collected from uninfected or LGTV-infected tick/N2a cells were resuspended in PBS and distributed into three groups (from the same preparations), that were either held as untreated group on ice, treated with Triton-X-100 (0.03%; 30 min, RT), or processed for three cycles of freezing at -80°C (for each freezing cycle samples were incubated for 1 h). After treatment and processing, protein lysates were prepared in a non-reducing and non-denaturating sample buffer (62.5 mM Tris-HCL, pH 6.8, 25% Glycerol and 1% Bromophenol blue), that maintained the proteins secondary structure and native charge density. Gels were pre-run for 60 min in gel running buffer (25 mM Tris and 192 mM Glycine). Uninfected or LGTV-infected exosomal preparations with different treatment or untreated samples were separated on 12% native-PAGE gels. Gels were transferred on to nitrocellulose membranes followed by immunoblotting using 4G2 or NS1 monoclonal antibodies followed by mouse monoclonal HRP-conjugated secondary antibodies (Santa Cruz Technologies, Inc). Total protein profiles (Coomassie blue stained gel) serve as loading controls. Antibody binding was detected with WesternBright ECL kit (Advansta, BioExpress). Blots were scanned using Chemidoc MP imaging system and instructions from the manufacturer (BioRad).

### ELISA

We collected N2a cell-derived exosomes from 5 x 10^6^ uninfected or LGTV-infected (MOI 6; 72 h p.i.) cells and resuspended in PBS (250 μl/sample). Exosomal fractions were grouped as untreated or treated with 0.1% of Triton-X-100 for 30 min. Nunc grade ELISA plates were coated with 50 μl of untreated or Triton-X-100 treated- uninfected or infected samples for overnight and incubated at 4°C. Samples were incubated with 4G2 antibody for 1 h, followed by HRP-conjugated mouse monoclonal secondary antibody for another 1 h as described [72]. We used SureBlue TMB Microwell Peroxidase substrate and Stop solution (KPL) and followed manufacturer’s instructions. After stopping the reactions with TMB Stop solution, optical density was measured from triplicate samples at an absorbance of 450nm using a Multimode infinite M200 Pro Microplate reader (Tecan).

### E-protein-antibody-beads binding assays

LGTV-infected tick or N2a cell-derived exosomes (from 72 h p.i.) were freshly isolated from 2 x 10^6^ tick cells (infected with MOI 1) or 5 x 10^5^ N2a cells (infected with 6 MOI). We also isolated exosomes from GW4869 inhibitor (5 μM) treated tick or N2a cells. For inhibitor treatment, cells were seeded in plates for overnight, changed to exosome-free FBS medium (in case of N2a cells) and after 4 h, treated with exosome release GW4869 inhibitor for 4h, followed by infection with LGTV for 72 h p.i. The exosomes collected from untreated or inhibitor treated cells were resuspended in PBS and grouped into three categories for both inhibitor treated or untreated samples as; untreated, treated with 4G2 antibody (that recognizes LGTV E-protein) or relevant isotype control antibody (R & D Systems) groups. Exosomal fractions were incubated for 1 h (RT) with respective antibodies followed by incubation (4°C) with protein A/G agarose beads (Pierce/ThermoScientific) for another 30 min. The antibody-beads complexes were spun (13k rpm) at 4°C for 30 min and supernatants were collected and lysed in RNA lysis buffer, processed for RNA extractions, followed by cDNA synthesis and QRT-PCR to detect LGTV loads.

### Transwell assays

Assays were performed to analyze the trans-migration of infectious exosomes from infected cells (seeded in inserts; upper chamber) to uninfected cells seeded in 12-well plates (lower chamber). Sterile, polycarbonate tissue culture-treated transwell inserts (12mm insert size) with 0.4 μm microporous membrane pore size were used in our assays (Corning). We plated, 1 x 10^5^ ISE6 tick or bEnd.3 cells in inserts (upper chamber) and 1 x 10^5^ HaCaT or N2a cells were seeded in 12-well plates (lower chamber). Inserts with tick or bEnd.3 cells were first kept in a separate 12-well plates containing 0.5 ml (in order to keep microporous membranes moist/wet) of L-15 (tick cells) or DMEM complete medium (bEnd.3 cells), respectively. Inhibitor-treated group in transwell assays was treated with 5 μM of GW4869 inhibitor, and at 24 h post treatment, tick cells or bEnd.3 cells were either infected with exosomes containing LGTV (25 μl of the exosome fraction collected from infected tick or bEnd.3 cells) or with LGTV from laboratory viral stocks (MOI 1 for tick cells or MOI 6 for bEnd.3 cells) prepared from infected Vero cell culture supernatants. Four hours post-infection, inserts with tick or bEnd.3 cells (with change of new media) were moved to 12-well plates containing HaCaT or N2a cells, respectively. Exosomes containing viral RNA and proteins produced from tick or bEnd.3 cells were allowed to transmigrate and infect HaCaT or N2a cells (that were kept uninfected). After, 48 h post incubation with inserts (containing either infected tick or bEnd.3 cells in inserts or upper chambers), HaCaT or N2a cells from lower chamber were washed with ice-cold PBS (3x) and collected for RNA extractions, cDNA synthesis and QRT-PCR to determine viral loads from cells.

### Plaque assays

Plaque assays were performed as described [72]. To determine infectious and replicative viruses after incubation with exosome and exosome-free supernatant fractions, we seeded Vero cells in 6-well plates at densities of 1 x 10^6^ cells per well, allowed them to adhere and grow as monolayers to reach 65–85% confluency (for ~24 h). Exosome fractions containing unknown PFU (plaque forming units) of LGTV viral genomes were collected from tick cells (5 x 10^6^ cells) or N2a cells or murine cortical neurons (1 x 10^5^ cells) and resuspended in 250 μl of PBS, 30 μl of this suspension (exosome fraction) was used for plaque assays. Exosome free supernatants (600 μl) that corresponds to the same ratio of exosome fractions were used as controls. Serial dilutions (1:10, 1:100 and 1:1000) of the exosomes or supernatant fractions were prepared in duplicate (shown are the representative plate images from two-three independent experiments). Monolayers of Vero cells were infected with exosomes or supernatant fractions or with exosomes from 1 μM or 10 μM (LGTV-infected N2a cells or mouse cortical neurons) of GW4869 inhibitor-treated or DMSO-treated controls. Four hours post infection, medium was removed and warm 2% Seaplaque agarose (Lonza) overlay with complete DMEM media (1:1 ratio) containing antibiotic and antimycotics solution (1% each; Sigma) was added. Plates were incubated for 6–7 days, at 37 **°**C, 5% CO2. After incubation period, plaques were stained with 0.03% of Neutral Red (Sigma) for 4 h, and the stain was removed to either count plaques on the same day or otherwise plates were incubated (inverted and covered in foil) for overnight, and then plaques were counted next day to determine the viral titers from LGTV-infected exosomal fractions from tick/neuronal cells.

### Isolation and infection of murine cortical neurons

Gestation period (day13) wild-type female C57BL/6 (Charles River Laboratories) mice were purchased and allowed to reacclimatize. All animal experiments were done in accordance with the University Animal Care and Use committee regulations. Primary cortical neurons were isolated from embryonic day-16 (E16) brains [72,73]. Murine cortical neurons (1 x 10^5^) were seeded in a 12-well plate coated with poly-L-Lysine and cultures were established in neurobasal complete medium with FBS. After 24h of plating, half of the medium was replaced with FBS-free neurobasal media, to slow growth of glial cells. For infection kinetics, cortical neurons were infected with LGTV (MOI 4) (after 48 h of post-seeding), neurons were collected at different time points (24, 48, 72 and 96 h p.i.) and processed for RNA extractions. For infection with neuronal cell-derived exosomes or supernatant fractions, 1 x 10^5^ murine cortical neurons were infected with 20 μl of neuronal exosomes (2.2 x 10^3^ pfu/ml) or 400 μl of exosome free supernatant fractions (collected from the step before PBS wash during exosome isolation, See **S2 Fig**). Cells were harvested at 48 h p.i. and processed for RNA extraction. Protein extractions were collected from uninfected or LGTV-infected cortical neurons (seeded at 1 x 10^7^ cells) or from exosomes isolated from these cell culture supernatants.

### Inhibitor studies

For exosome inhibition studies, we used GW4869 a cell permeable, selective inhibitor for Neutral Sphingomyelinase (N-SMase) (Santa Cruz Biotechnologies, Inc) and DMSO as controls. Cells did not show any toxicity at tested doses. For both transwell assays, inhibitor-treated group was treated with 5 μM exosome inhibitor. N2a cells or murine cortical neurons were seeded at 1 x 10^5^ cells in a 12-well plate. Next day, before treatment with inhibitor, cells were replaced with bovine exosome free-FBS (Systems BioSciences, Inc.) containing DMEM (N2a cells) or neurobasal medium (neurons). Cells were treated with either 1, 5 or 10 μM (N2a cells) or with 1, 10 or 20 μM (neurons) of inhibitor for 4 h, followed by infection with LGTV (N2a cells, MOI 6; cortical neurons MOI 4). Plaque assays were performed with 30 μl of exosome fractions derived from N2a cells or cortical neurons to determine the unknown titers for both DMSO control or inhibitor treated groups, respectively. N2a cells were either pre- or post- treated with inhibitor, where cells were first treated with inhibitor (5 μM) for 4 h followed by infection for 72 h or vice versa, respectively. Supernatants collected from uninfected controls and cells infected with LGTV (laboratory virus stocks with known titers) (48 h p.i.) were processed for exosome isolation. Purified exosomes were resuspended in PBS and processed for either RNA extraction or used for infection of new cells to determine re-infection kinetics or used to determine viral titers by plaque assays. For GW4869 treatment on laboratory virus stocks, we treated the viral supernatants (collected from Vero cells) with known titers (7.4 x 10^6^ pfu/ml). We used 30 μl of the virus stocks and treated with either DMSO or inhibitor (5 and 10 μM for 4 h at 37°C) followed by immunoblotting with 4G2 antibody. For 4G2 functional blocking antibody studies, we plated N2a cells (2 x 10^5^), treated with 5 μg of antibody for 4 h and infected cells with freshly isolated exosomes from LGTV-infected (72 h p.i.) N2a cells. N2a cells were infected through infectious exosomes for 72 h p.i. and collected for RNA extractions and QRT-PCR analysis. Untreated samples serve as control. For Pitstop-2 inhibitor treatment, N2a cells (2 x 10^5^) were treated with 30 μM Pitstop-2 (dissolved in DMSO) for 15 min followed by infection through freshly isolated exosomes from LGTV-infected (72 h p.i.) N2a cells. Cells were collected for RNA extractions after 72 h p. i. and further processed for QRT-PCR. DMSO treated cells served as controls.

### Statistics

Statistical difference observed in data sets was analyzed using GraphPad Prism6 software and Microsoft Excel. The non-paired, two-tail Student *t* test was performed (for data to compare two means) for the entire analysis. Error bars represent mean (+SD) values, P values of <0.05 were considered significant in all analysis. Statistical test and P values are indicated for significance.

### Ethics statement

All animal work in this study was carried out in strict accordance with the recommendations in the Guide for the Care and Use of Laboratory Animals of the National Institute of Health. The approved protocol from the Institutional Animal Care and Use Committee (Animal Welfare Assurance Number: A3172-01) was used in this study (permit number: 16–017).

## Supporting information

S1 Fig LGTV infection kinetics in ISE6 tick cells and Vero cells. QRT-PCR analysis showing levels of LGTV in tick cells (A) or Vero cells (B) at different time points (24, 48, 72 h p.i). 1 x 10^5^ tick or Vero cells were infected with either 1 or 6 MOI of LGTV, respectively. UI indicates uninfected and I indicates LGTV-infected. Representative data is shown from at least three independent experiments. P value determined by Student’s two-tail *t* test is shown. (C) Schematic representation of DG-Exos using density gradients of iodixanol is shown.
(TIF)

S2 Fig Schematic representation of the method used for exosomes isolation from different cells. The method for exosomes isolation was adapted from [29]. Culture supernatant/fluid was spun at 300 g for 10 min to remove floating cells and supernatant fraction was collected. Supernatant was spun at 2000 g for 10 min to remove dead cells and resulting supernatant fraction was collected and spun again at 10, 000 g for 30 min to remove any remaining cell debris. The supernatant fraction collected from the previous step was spun at 100, 000 g for 70 min (for bEnd.3 cells, N2a cells, cortical neurons) or for 155 min (for tick cells) in an ultracentrifugation unit. Supernatants resulted after the above longer spin step were used in all the experiments as supernatant fractions. The exosomes containing pellet fraction was washed in ice-cold PBS and spun at 100, 000 g for 70 min (for bEnd.3 cells, N2a cells or cortical neurons) or for 155 min (for tick cells). The pellet resulted after this wash is considered as exosome fraction in all the experiments. The exosome pellet/fraction was either dissolved in PBS (for performing re-infection, plaque or transwell assays, Native PAGE and 4G2-antibody-beads-binding assay), or in RNA lysis buffer (for total RNA extractions) or in modified RIPA buffer for protein extractions.
(TIF)

S3 Fig Presence of LGTV RNA in exosomes isolated from infected-ISE6 tick cells grown in exosome-free FBS media and infection kinetics and re-infection in HaCaT or HUVEC cells. QRT-PCR analysis showing copy number of LGTV RNA (A) or LGTV total loads (B) in exosomes isolated from tick cells at 72 h p.i. (5 x 10^6^ tick cells infected with 1 MOI of LGTV), cells were grown in commercially available bovine exosome-free FBS medium. LGTV transcript levels were normalized to tick beta-actin. (C) Immunoblot gel image showing levels of E-protein or total protein loads (in Ponceau stained image) in LGTV-infected tick cell-derived exosomes treated with proteinase K (50 μg/ml, 15 min, 37°C) is shown. The uninfected-untreated or treated groups serve as control. Plaque assays performed with different dilutions (1:10, 1:100, 1:1000) of exosomes fraction (D) or corresponding different volumes (600, 60, 6 μl) of supernatant fractions (E) prepared from tick cells is shown. Ruler at the top determines scale for the represented plaque assays from three independent experiments. (F) QRT-PCR analysis showing levels of LGTV in HaCaT cells at different time points (24, 48 and 72 h p.i.). LGTV (6 MOI) was used to infect 1 x 10^5^ HaCaT cells. (G) Viral re-infection kinetics as determined by the presence of LGTV in HaCaT cells (1 x 10^5^ cells at 24, 48 and 72 h p.i.) infected by treatment with exosome (20 μl) or supernatant (400 μl) fractions prepared from 72 h p.i. LGTV-infected tick cells that were grown in Exosome-free FBS medium are shown. (H) QRT-PCR analysis showing levels of LGTV in HUVEC cells at different time points (24, 48, 72 h p.i.). UI indicates uninfected and I indicates LGTV-infected. (I) Infection of HUVEC cells with infectious tick cell-derived exosomes or supernatant fractions showing LGTV loads at 48 h p.i. is presented. LGTV transcript levels in HaCaT and HUVEC cells were normalized to human beta-actin. P value determined by Student’s two-tail *t* test is shown. Representative data is shown from two independent experiments.
(TIF)

S4 Fig LGTV infection kinetics in bEnd.3 and N2a cells. QRT-PCR analysis showing levels of LGTV in bEnd.3 cells (A, B) or copy numbers (C) at different time points (24, 48, 72 h p.i, respectively). Infection kinetics at later time points (96 and 120 h p.i.) is shown for bEnd.3 cells (B). (D) Infection kinetics with increasing LGTV loads in N2a cells is shown. Six MOI of LGTV virus stock was used to infect 1 x 10^5^ bEnd.3 or N2a cells. UI indicates uninfected and I indicates LGTV-infected. LGTV transcript levels in bEnd.3 and N2a cells were normalized to mouse beta-actin, respectively. Representative data is shown from at least three independent experiments. P value determined by Student’s two-tailed *t* test is shown.
(TIF)

S5 Fig Detection of LGTV RNA in exosomes isolated from N2a cells using commercially available exosome isolation reagent. (A) Schematic representation of exosome isolation using commercial kit and manufacturer’s protocol is shown. (B) Cryo-EM images showing exosomes isolated using the kit protocol from uninfected and LGTV-infected (MOI 6; 72 h p.i.), N2a cells (1 x 10^7^). (C) OptiPrep DG-isolated fractions from laboratory virus supernatants as stocks showing E-protein. The data from Fig 3E for E-protein levels in exosomal fractions is included for comparison. QRT-PCR analysis showing levels of LGTV (D) in exosomes isolated from N2a cells at different time points (24, 48 and 72 h p.i.) using commercially available exosome isolation reagent. LGTV transcript levels were normalized to mouse beta-actin. P value determined by Student’s two-tail *t* test is shown. (E) Immunoblot showing E-protein in de-glycosylated form from laboratory virus stocks. The exosomal fraction with E-protein at similar size is shown for comparison. (F) QRT-PCR showing viral loads in LGTV-infected N2a cell-derived exosomes treated with Triton-X-100, followed by RNaseA. (G) LGTV-infected N2a cell-derived exosomes treated with proteinase K (100 μg/μl, 15 min, 37°C) is shown. The uninfected but untreated groups serve as control.
(TIF)

S6 Fig Detection of viral plaques and LGTV reinfection kinetics using exosomes derived from N2a cells. Plaque assays performed with different dilutions (1:10, 1:100, 1:1000) of exosome pellet (A) or corresponding different volumes (600, 60, 6 μl) of supernatant fractions (B) prepared from LGTV-infected N2a cells is shown. Ruler at the top determines the scale for the plaque assay from the representative images (three independent experiments). (C) QRT-PCR analysis of the infection of naïve neuronal N2a cells (1 x 10^5^ cells) collected at 24 h p.i. with exosome (20 μl) or supernatant (same ratio of exosome fraction) fractions prepared from LGTV-infected N2a cells (from 24, 48 and 72 h p.i.) or uninfected cells show presence of LGTV RNA in infected cells. LGTV transcript levels were normalized to mouse beta-actin. P value determined by Student’s two-tail *t* test is shown.
(TIF)

S7 Fig Detection of viral plaques using exosomes derived from mouse cortical neurons. Plaque assays performed with different dilutions (1:10, 1:100, 1:1000) of exosome pellet (A) or corresponding different volumes (600, 60, 6 μl) of supernatant fractions (B) prepared from LGTV-infected cortical neuronal cells is shown. Ruler at the top determines the scale for the plaque assay from the representative images. Representative images from two independent experiments are shown.
(TIF)
