# Supplementary figures and images for: Exosomes serve as novel modes of tick-borne flavivirus transmission from arthropod to human cells and facilitates dissemination of viral RNA and proteins to the vertebrate neuronal cells
Source: PLoS Pathog. 2018 Jan 4;14(1):e1006764. doi: 10.1371/journal.ppat.1006764 (PMC5754134; doi:10.1371/journal.ppat.1006764)

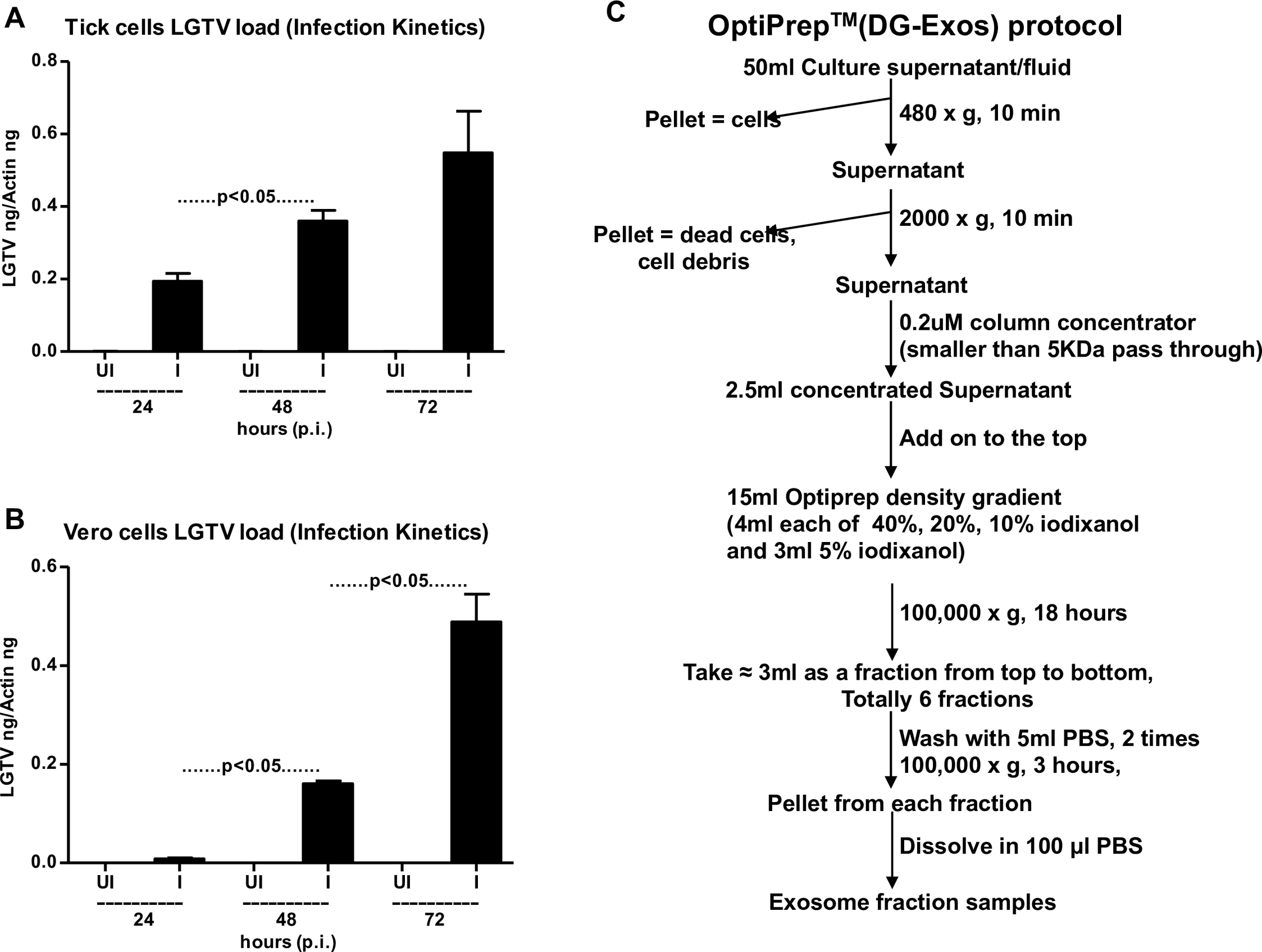

Supplement: S1 Fig — QRT-PCR analysis showing levels of LGTV in tick cells (A) or Vero cells (B) at different time points (24, 48, 72 h p.i). 1 x 10^5^ tick or Vero cells were infected with either 1 or 6 MOI of LGTV, respectively. UI indicates uninfected and I indicates LGTV-infected. Representative data is shown from at least three independent experiments. P value determined by Student’s two-tail t test is shown. (C) Schematic representation of DG-Exos using density gradients of iodixanol is shown. (TIF) [file ppat.1006764.s001.tif]

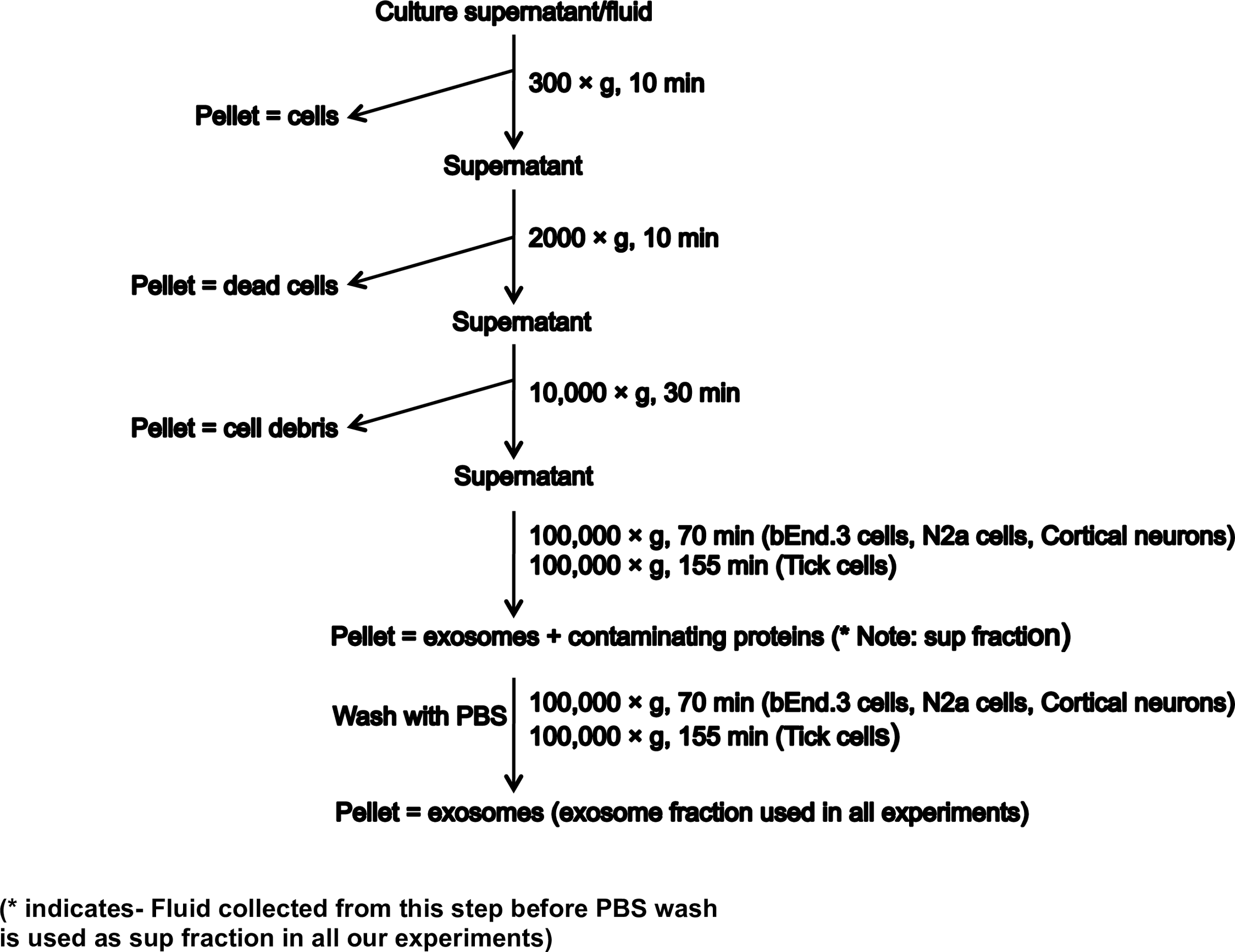

Supplement: S2 Fig — The method for exosomes isolation was adapted from [29]. Culture supernatant/fluid was spun at 300 g for 10 min to remove floating cells and supernatant fraction was collected. Supernatant was spun at 2000 g for 10 min to remove dead cells and resulting supernatant fraction was collected and spun again at 10, 000 g for 30 min to remove any remaining cell debris. The supernatant fraction collected from the previous step was spun at 100, 000 g for 70 min (for bEnd.3 cells, N2a cells, cortical neurons) or for 155 min (for tick cells) in an ultracentrifugation unit. Supernatants resulted after the above longer spin step were used in all the experiments as supernatant fractions. The exosomes containing pellet fraction was washed in ice-cold PBS and spun at 100, 000 g for 70 min (for bEnd.3 cells, N2a cells or cortical neurons) or for 155 min (for tick cells). The pellet resulted after this wash is considered as exosome fraction in all the experiments. The exosome pellet/fraction was either dissolved in PBS (for performing re-infection, plaque or transwell assays, Native PAGE and 4G2-antibody-beads-binding assay), or in RNA lysis buffer (for total RNA extractions) or in modified RIPA buffer for protein extractions. (TIF) [file ppat.1006764.s002.tif]

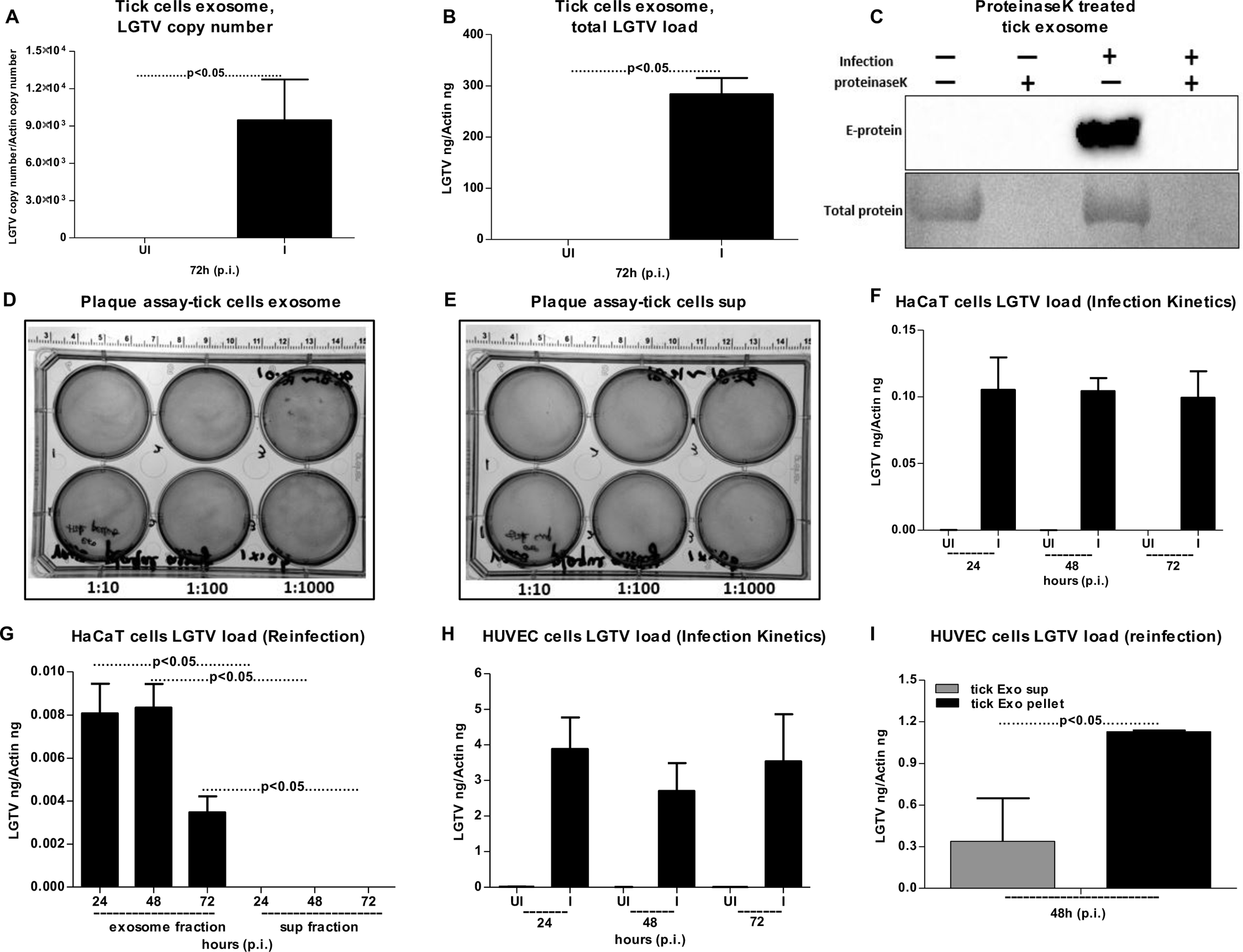

Supplement: S3 Fig — QRT-PCR analysis showing copy number of LGTV RNA (A) or LGTV total loads (B) in exosomes isolated from tick cells at 72 h p.i. (5 x 10^6^ tick cells infected with 1 MOI of LGTV), cells were grown in commercially available bovine exosome-free FBS medium. LGTV transcript levels were normalized to tick beta-actin. (C) Immunoblot gel image showing levels of E-protein or total protein loads (in Ponceau stained image) in LGTV-infected tick cell-derived exosomes treated with proteinase K (50 μg/ml, 15 min, 37°C) is shown. The uninfected-untreated or treated groups serve as control. Plaque assays performed with different dilutions (1:10, 1:100, 1:1000) of exosomes fraction (D) or corresponding different volumes (600, 60, 6 μl) of supernatant fractions (E) prepared from tick cells is shown. Ruler at the top determines scale for the represented plaque assays from three independent experiments. (F) QRT-PCR analysis showing levels of LGTV in HaCaT cells at different time points (24, 48 and 72 h p.i.). LGTV (6 MOI) was used to infect 1 x 10^5^ HaCaT cells. (G) Viral re-infection kinetics as determined by the presence of LGTV in HaCaT cells (1 x 10^5^ cells at 24, 48 and 72 h p.i.) infected by treatment with exosome (20 μl) or supernatant (400 μl) fractions prepared from 72 h p.i. LGTV-infected tick cells that were grown in Exosome-free FBS medium are shown. (H) QRT-PCR analysis showing levels of LGTV in HUVEC cells at different time points (24, 48, 72 h p.i.). UI indicates uninfected and I indicates LGTV-infected. (I) Infection of HUVEC cells with infectious tick cell-derived exosomes or supernatant fractions showing LGTV loads at 48 h p.i. is presented. LGTV transcript levels in HaCaT and HUVEC cells were normalized to human beta-actin. P value determined by Student’s two-tail t test is shown. Representative data is shown from two independent experiments. (TIF) [file ppat.1006764.s003.tif]

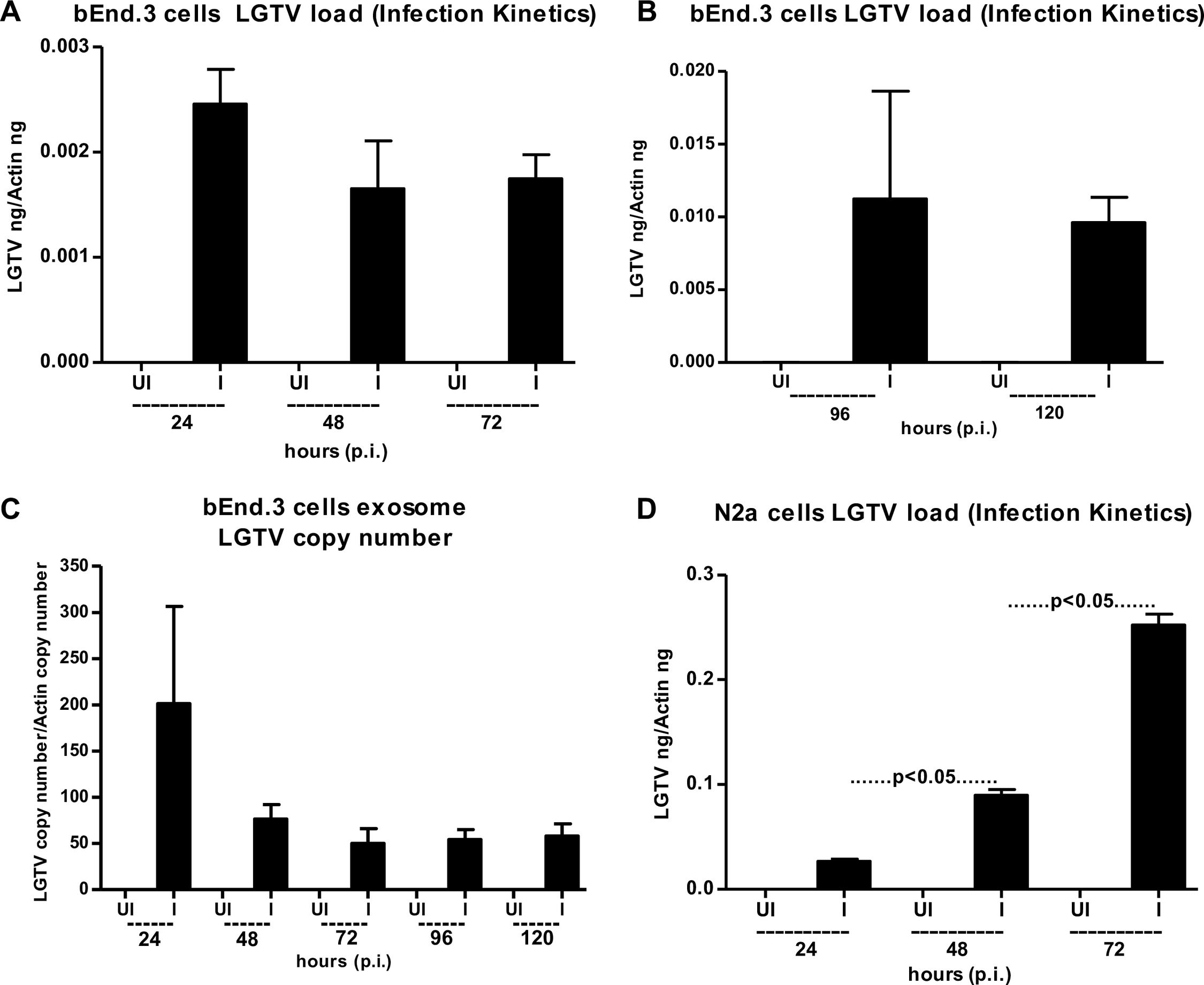

Supplement: S4 Fig — QRT-PCR analysis showing levels of LGTV in bEnd.3 cells (A, B) or copy numbers (C) at different time points (24, 48, 72 h p.i, respectively). Infection kinetics at later time points (96 and 120 h p.i.) is shown for bEnd.3 cells (B). (D) Infection kinetics with increasing LGTV loads in N2a cells is shown. Six MOI of LGTV virus stock was used to infect 1 x 10^5^ bEnd.3 or N2a cells. UI indicates uninfected and I indicates LGTV-infected. LGTV transcript levels in bEnd.3 and N2a cells were normalized to mouse beta-actin, respectively. Representative data is shown from at least three independent experiments. P value determined by Student’s two-tailed t test is shown. (TIF) [file ppat.1006764.s004.tif]

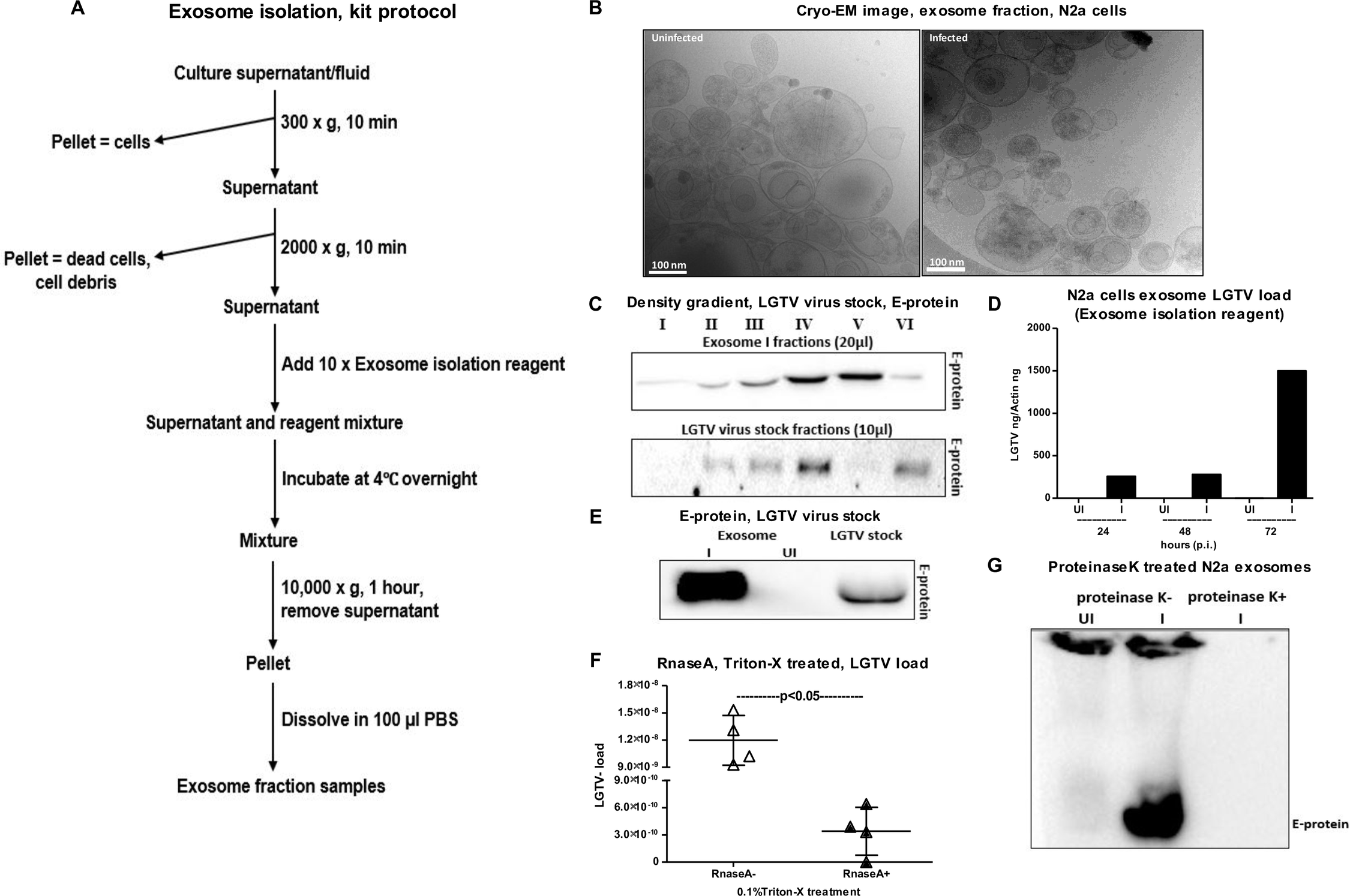

Supplement: S5 Fig — (A) Schematic representation of exosome isolation using commercial kit and manufacturer’s protocol is shown. (B) Cryo-EM images showing exosomes isolated using the kit protocol from uninfected and LGTV-infected (MOI 6; 72 h p.i.), N2a cells (1 x 10^7^). (C) OptiPrep DG-isolated fractions from laboratory virus supernatants as stocks showing E-protein. The data from Fig 3E for E-protein levels in exosomal fractions is included for comparison. QRT-PCR analysis showing levels of LGTV (D) in exosomes isolated from N2a cells at different time points (24, 48 and 72 h p.i.) using commercially available exosome isolation reagent. LGTV transcript levels were normalized to mouse beta-actin. P value determined by Student’s two-tail t test is shown. (E) Immunoblot showing E-protein in de-glycosylated form from laboratory virus stocks. The exosomal fraction with E-protein at similar size is shown for comparison. (F) QRT-PCR showing viral loads in LGTV-infected N2a cell-derived exosomes treated with Triton-X-100, followed by RNaseA. (G) LGTV-infected N2a cell-derived exosomes treated with proteinase K (100 μg/μl, 15 min, 37°C) is shown. The uninfected but untreated groups serve as control. (TIF) [file ppat.1006764.s005.tif]

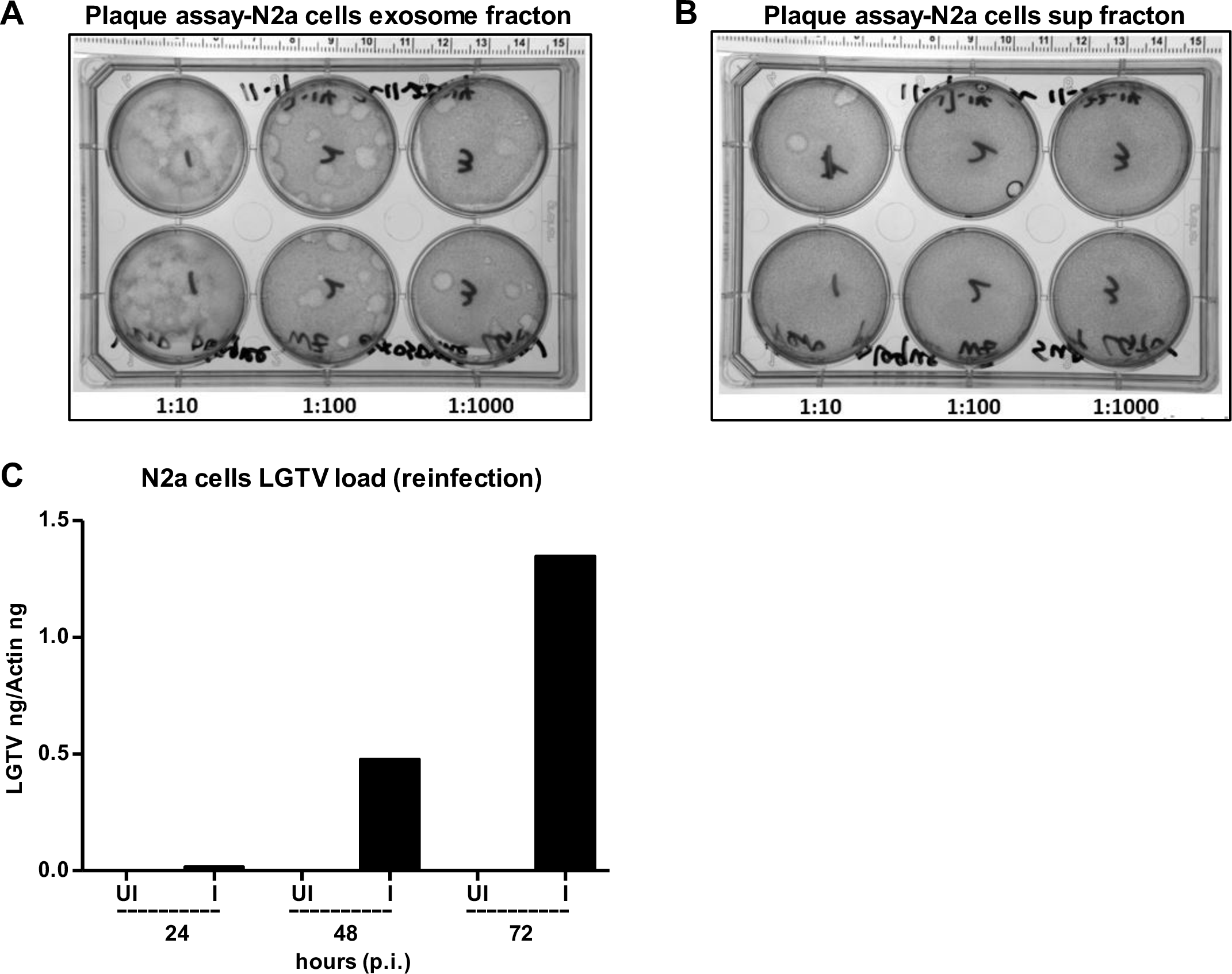

Supplement: S6 Fig — Plaque assays performed with different dilutions (1:10, 1:100, 1:1000) of exosome pellet (A) or corresponding different volumes (600, 60, 6 μl) of supernatant fractions (B) prepared from LGTV-infected N2a cells is shown. Ruler at the top determines the scale for the plaque assay from the representative images (three independent experiments). (C) QRT-PCR analysis of the infection of naïve neuronal N2a cells (1 x 10^5^ cells) collected at 24 h p.i. with exosome (20 μl) or supernatant (same ratio of exosome fraction) fractions prepared from LGTV-infected N2a cells (from 24, 48 and 72 h p.i.) or uninfected cells show presence of LGTV RNA in infected cells. LGTV transcript levels were normalized to mouse beta-actin. P value determined by Student’s two-tail t test is shown. (TIF) [file ppat.1006764.s006.tif]

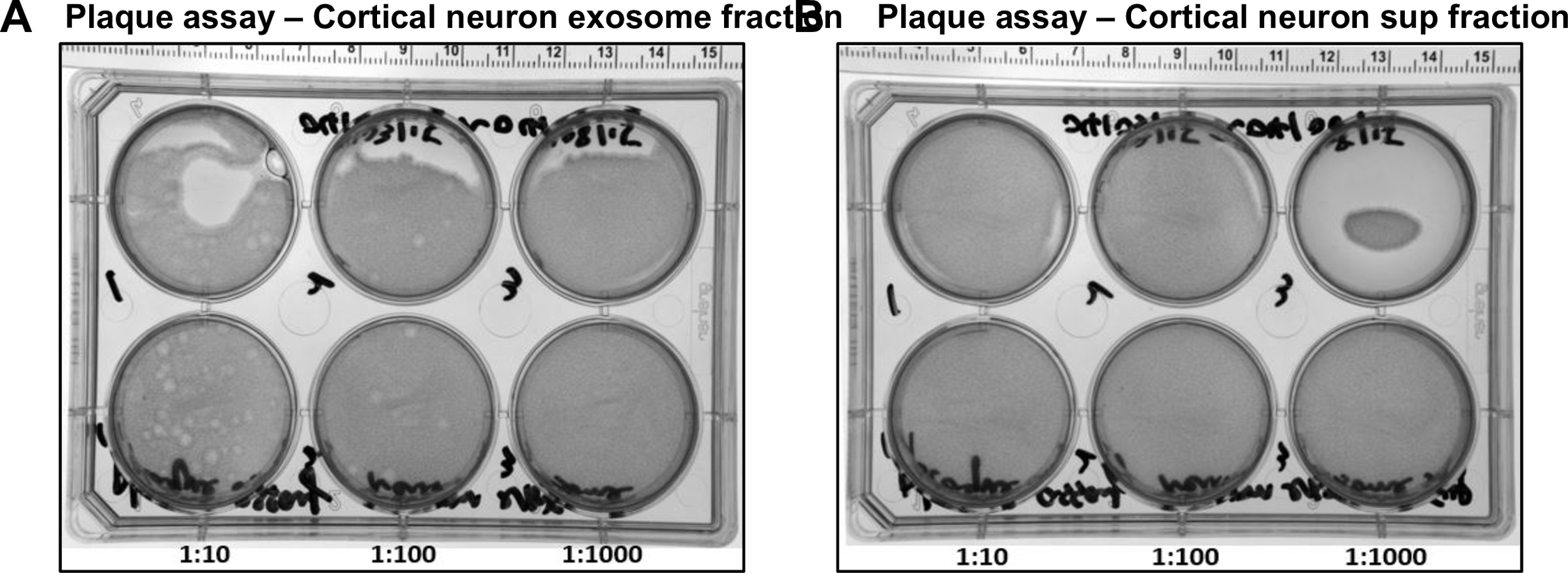

Supplement: S7 Fig — Plaque assays performed with different dilutions (1:10, 1:100, 1:1000) of exosome pellet (A) or corresponding different volumes (600, 60, 6 μl) of supernatant fractions (B) prepared from LGTV-infected cortical neuronal cells is shown. Ruler at the top determines the scale for the plaque assay from the representative images. Representative images from two independent experiments are shown. (TIF) [file ppat.1006764.s007.tif]
